# The dietary isothiocyanate sulforaphane modulates gene expression and alternative gene splicing in a PTEN null preclinical murine model of prostate cancer

**DOI:** 10.1186/1476-4598-9-189

**Published:** 2010-07-13

**Authors:** Maria H Traka, Caroline A Spinks, Joanne F Doleman, Antonietta Melchini, Richard Y Ball, Robert D Mills, Richard F Mithen

**Affiliations:** 1 Plant Natural Products and Health Programme, Institute of Food Research, Norwich, NR4 7UA, UK; 2 Pharmaco-Biological Department, School of Pharmacy, University of Messina, Messina 98168, Italy; 3 Norfolk and Waveney Cellular Pathology Network, Norfolk and Norwich University Hospital, Colney Lane, Norwich, NR4 7UB, UK; 4 Department of Urology, Norfolk and Norwich University Hospital, Colney Lane, Norwich, NR4 7UY, UK

## Abstract

**Background:**

Dietary or therapeutic interventions to counteract the loss of PTEN expression could contribute to the prevention of prostate carcinogenesis or reduce the rate of cancer progression. In this study, we investigate the interaction between sulforaphane, a dietary isothiocyanate derived from broccoli, PTEN expression and gene expression in pre malignant prostate tissue.

**Results:**

We initially describe heterogeneity in expression of PTEN in non-malignant prostate tissue of men deemed to be at risk of prostate cancer. We subsequently use the mouse prostate-specific PTEN deletion model, to show that sulforaphane suppresses transcriptional changes induced by PTEN deletion and induces additional changes in gene expression associated with cell cycle arrest and apoptosis in PTEN null tissue, but has no effect on transcription in wild type tissue. Comparative analyses of changes in gene expression in mouse and human prostate tissue indicate that similar changes can be induced in humans with a broccoli-rich diet. Global analyses of exon expression demonstrated that sulforaphane interacts with PTEN deletion to modulate alternative gene splicing, illustrated through a more detailed analysis of DMBT1 splicing.

**Conclusion:**

To our knowledge, this is the first report of how diet may perturb changes in transcription induced by PTEN deletion, and the effects of diet on global patterns of alternative gene splicing. The study exemplifies the complex interaction between diet, genotype and gene expression, and the multiple modes of action of small bioactive dietary components.

## Background

Prostate cancer, one of the more common neoplasms in the western world, arises through the progressive development of one or more pre neoplastic lesions into adenocarcinoma, and subsequently to metastatic disease. Recent advances have identified key genetic alterations that can initiate prostate carcinogenesis, and enhance the probability of cancer progression. Foremost amongst these is the deletion or inactivation of the PTEN tumour suppressor gene, an antagonist of the phosphatidylinositol-3-kinase (PI3K/AKT) signaling pathway that promotes cell survival and proliferation. PTEN deletion in an epithelial stem cell can be an early initiating event leading to prostatic intraepithelial neoplasia (PIN), and subsequently to cancer [1,2]. Thus, heterogeneity in expression of PTEN in the aging prostate tissue may lead to the development of multifocal pre invasive lesions. Therapeutic and dietary approaches to target prostate cells with PTEN deletion and hyperactivated PI3K/AKT signaling may make a major contribution to reducing the incidence and progression of prostate cancer.

Isothiocyanates such as sulforaphane [SF; (-)-1-isothiocyanato-(4*R*)-methylsulfinylbutane] have been shown to reduce prostate tumour growth and pulmonary metastasis in the TRAMP mouse model of prostate cancer [3,4], and to reduce the growth of prostate cancer xenografts in immune-deficient mice derived from the PTEN-deficient PC3 metastatic cell line [5]. Isothiocyanates have been shown to exhibit several potential chemoprotective activities in cell and animal models [6,7], including the partial suppression of pAKT expression [3,8]. The biological activity of isothiocyanates may also provide an explanation for the inverse correlation between diets rich in cruciferous vegetables such as broccoli (the major source of SF in the diet) and the incidence and progression of prostate cancer found in both case control and prospective epidemiological studies [9-12]. Moreover, in a recent human intervention study it was shown that a diet rich in broccoli resulted in changes in gene expression associated with insulin and EGF signaling in prostate tissue of men who had been diagnosed with high grade PIN (HGPIN) [13], suggesting a potential effect of sulforaphane on PI3K/AKT signaling in humans. Thus, dietary isothiocyanates may be potential candidates to target cells with PTEN deletion or inactivation and enhanced pAKT expression in pre-cancerous prostate tissue.

In the current study, we initially show that that there is significant variation in PTEN and pAKT expression in non-neoplastic tissue of men who had previously been diagnosed with HGPIN. We then demonstrate that SF has differential effects on the viability and proliferation of human cell lines that differ in PTEN expression. We additionally report with the use of PTEN^L/L^;PB-Cre4 mice [14], that dietary intervention with SF has no effect on gene expression in mouse prostate tissue with PTEN expression, whereas in isogenic PTEN-deficient tissue SF acts to attenuate and reverse changes in PTEN deletion-mediated gene expression and induces additional changes in gene expression. We also show that there is a significant overlap in changes in gene expression induced by SF in PTEN null prostate tissue of mice with that induced in prostate tissue of men consuming a broccoli-rich diet. Finally, through the use of exon arrays, we find that SF interacts with PTEN deletion to both attenuate and promote alternative gene splicing. Our results support the finding that cells that have PTEN deletion and associated activation of PI3K/AKT signaling are hypersensitive to SF. It is possible that this leads to these cells being less competitive in growth compared to cells with wild type PTEN expression and provides an explanation of how consuming broccoli can reduce the risk of prostate cancer incidence and progression. In addition, it suggests potential therapeutic applications for sulforaphane.

## Results

### Heterogeneity of expression of PTEN and pAKT in non-neoplastic tissue from men diagnosed with HGPIN

**Figure 1:**
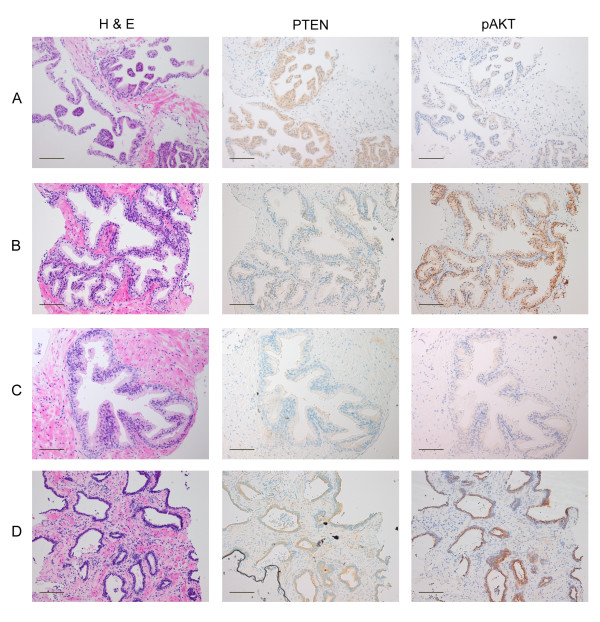
**Heterogeneity in PTEN and pAKT expression in non-malignant human prostate tissue**. Representative examples of non-malignant human prostate tissue illustrating heterogeneity of PTEN and pAKT expression: (A) illustrates high level of nuclear and cytoplastic expression of PTEN with no pAKT expression, (B) illustrates loss of PTEN expression with enhanced pAKT expression, (C) illustrates loss of PTEN expression without apparent increase in pAKT, (D) illustrates expression of both PTEN and pAKT associated with atrophy. Bar = 100 μm.

**Figure 2:**
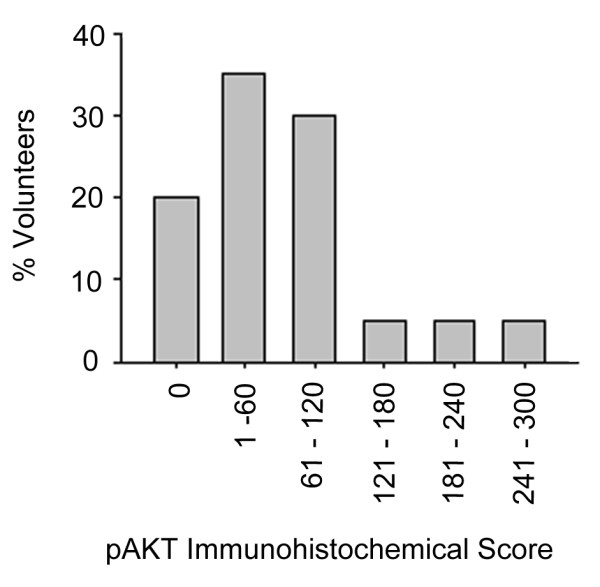
**Variation in pAKT expression in non-malignant tissue of 22 men who had previously received a diagnosis of HGPIN**. The immmunohistochemical score (Q) of tissue samples was determined by estimating the percentage of epithelial cells positively stained for pAKT (P) and multiplying it by the intensity of the staining (I).Weak staining was scored 1, moderate staining scored 2, and strong staining scored 3 to give a possible maximum score of 300 (Q = P × I). The data were divided into sextiles and the frequency (%) of volunteers in each sextile is shown here.

**Figure 3:**
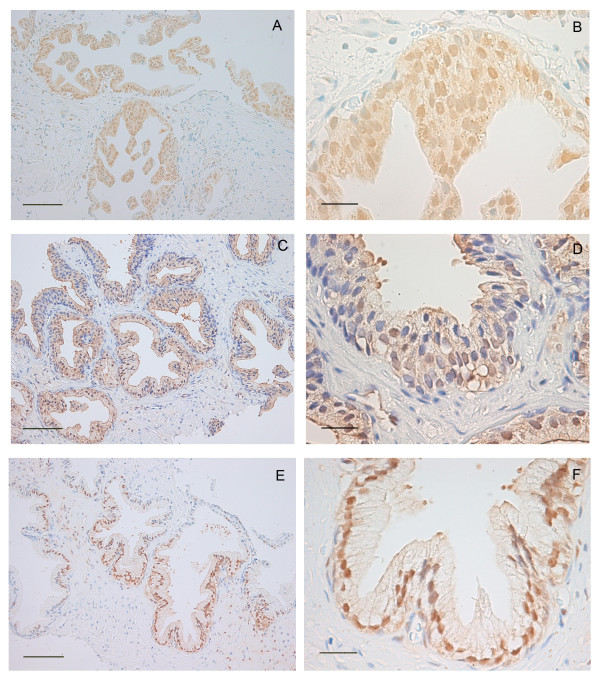
**Localisation of PTEN expression in non-neoplastic human prostate tissue**. (**A **and **B**) Nuclear and cytoplasmic expression of PTEN, (**C **and **D**) cytoplasmic but lack of nuclear expression of PTEN, (**E **and **F**) high level of nuclear expression of PTEN with reduced levels of cytoplasmic expression. Bars = 100 μm (**A**, **C **and **E**), 30 μm (**B**, **D **and **F**).

We obtained TRUS-guided prostate biopsy tissue from 21 volunteers who had previously received a diagnosis of HGPIN (mean time since diagnosis 135 ± 107 days, mean age 64 ± 4.3 years), and investigated PTEN and pAKT expression via immunohistochemichal staining in non-neoplastic prostate tissue. We found extensive heterogeneity in pAKT expression both among and within individuals (Figures 1 and 2). We also found extensive variation in expression of PTEN, with cells having both nuclear and cytoplasmic expression, solely cytoplasmic expression or neither nuclear nor cytoplasmic expression (Figures 1 and 3). While intense pAKT expression was associated with lack of both nuclear and cytoplasmic PTEN expression, a close inverse association between pAKT and PTEN expression was not apparent. In particular, epithelial cells from atrophic glands often had both pAKT and PTEN expression (Figure 1).

### The dietary isothiocyanate sulforaphane inhibits the growth of the cancerous PTEN-deficient PC3 prostate cells at lower concentrations than the non-cancerous PNT1A cell line

**Figure 4:**
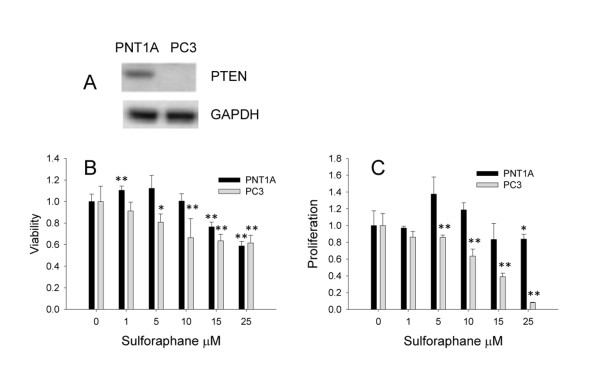
**Viability and proliferation of PNT1A and PC3 human prostate cells**. **A**, Expression of PTEN in PNT1A and PC3 cells. **B**, Viability of PNT1A and PC3 cells in response to 24 h treatment with SF relative to control measured by WST-1 assay. **C**, Proliferation of PNT1A and PC3 cells in response to 24 h treatment with SF relative to control measured by ELISA following BrdU incorporation. * p ≤ 0.05 **p ≤ 0.001.

As an initial investigation into the potential interaction between PTEN deletion and SF, we quantified the effect of SF on the growth and proliferation of a human cancerous prostate cell line, PC3, which is deficient in PTEN and has high constitutive expression of pAKT [15], compared to the non-cancerous cell line PNT1A, which expresses PTEN and a low constitutive expression of pAKT. We found that at low concentrations, SF enhanced the growth and proliferation of PNTIA cells, while inhibited the growth and proliferation of PC3 cells. Both cell lines were inhibited at higher supraphysiological concentrations of SF (Figure 4).

### SF has differential effects on gene expression in mouse prostate tissue that varies in PTEN expression

To explore the interaction between PTEN expression and activity of SF specifically, we used PTEN^L/L^;PB-Cre4 mice. In this model, segregation of Cre within a backcross generation results in PTEN deficient ('null') and wild type (WT) PTEN mice within the same litter, enabling the specific effects of PTEN deletion to be investigated [14]. We supplemented the diet of PTEN null mice and their WT littermates with either 0.1 μMol SF/g diet (low SF diet, LSF) or 1 μMol SF/g diet (high SF diet, HSF) and compared it to mice fed a control diet without added SF.

**Figure 5:**
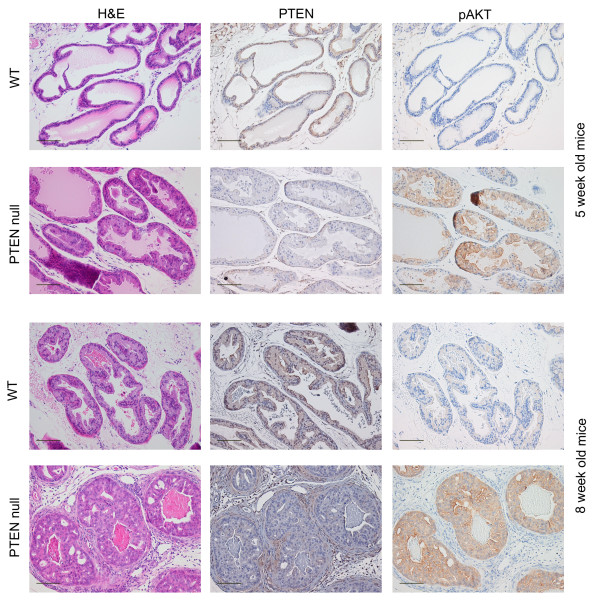
**Representative sections of mouse prostate illustrating effect of PTEN deletion on expression of PTEN and pAKT**. Bar = 100 μm.

**Figure 6:**
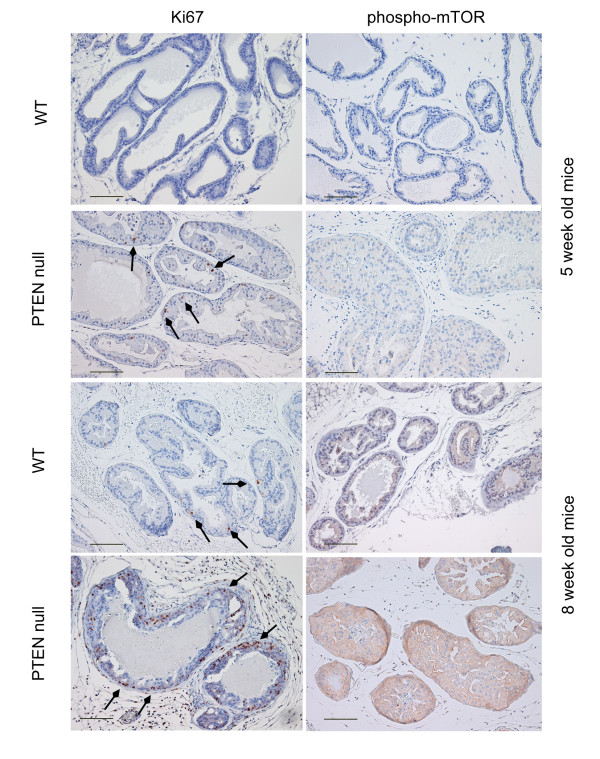
**Representative sections of mouse prostate illustrating effect of PTEN deletion on expression of Ki67 (arrows) and phosphorylated mTOR (red staining)**. Bar = 100 μm.

WT mice had normal prostate histology. However, at eight weeks foci of mild epithelial hyperplasia were observed in some sections, although they were not significantly associated with diet. PTEN deletion at both five and eight weeks was confirmed through RT-PCR analyses of prostate tissue (data not shown) and immunohistochemichal staining (Figure 5). Histopathological development of murine prostate intraepithelial neoplasia (mPIN) due to PTEN deletion was very similar to that previously described [14]. PTEN null prostate tissue from five-week old mice exhibited epithelial hyperplasia and some foci with early indication of mPIN which also showed increased pAKT expression. This association was more prominent at eight-weeks, where pAKT was highly expressed in all epithelial cells, with most glands exhibiting mPIN. Ki67 was widely expressed at both five and eight weeks in PTEN null prostate tissue but expression of phosphorylated mTOR was only detected at eight weeks (Figure 6). Diet did not have an obvious effect on the appearance of mPIN or expression of these proteins.

**Figure 7:**
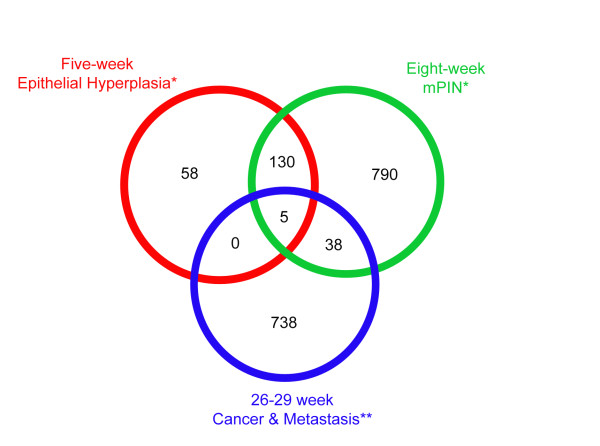
**Overlap of PTEN signature genes (restricted to those with a Unigene designation) changed after 5 and 8 weeks and after 26-29 weeks**. *this study; **data from Wang et al. (2003).

PTEN deletion resulted in multiple changes in gene expression at both five weeks and eight weeks (Table 1), associated with epithelial hyperplasia and mPIN, respectively. At five weeks, the PTEN deletion resulted in changes in expression of 198 transcripts (adjusted p < 0.05), while at eight weeks the deletion resulted in changes in expression of 989 transcripts (Table 1, Additional file 1 - S1). These are designated '5-week and 8-week PTEN signature genes'. 135 transcripts were common between these two lists of genes, which was significantly greater than the 11 transcripts that would have been expected by chance (Fishers exact test, p = 5.8 × 10^-44^). A previous study reported changes in gene expression in the same mouse model between wild type prostate tissue and invasive adenocarcinoma in PTEN null prostate tissue obtained 26-29 weeks after birth, in which there were changes in expression of 1041 out of a possible 10290 genes/ESTs that were present on a custom-made cDNA array [14]. We compared this list with our 5-week and 8-week PTEN signature gene lists for which we had Unigene designations (193 and 963 respectively), but found little overlap between either the 5 week PTEN signature list or the 8 week PTEN signature list with the gene list obtained from analyses of tissue after 26-29 weeks (Figure 7). The five genes that were in common in all three lists are chemokine (C-C motif) ligand 20 (Ccl20), extracellular proteinase inhibitor (Expi), G protein-coupled receptor family C group 5 member A (Gprc5a), CD24a antigen (Cd24a) and calcium and integrin binding 1 (calmyrin, Cib1). It is therefore likely that PTEN deletion initiates a transcriptional cascade, in which genes that are altered in their expression in the early phase are distinct from that in the later phases associated with metastatic disease.

**Table 1:** Changes in transcript expression in PTEN null and WT mice following SF-rich diets (LSF, HSF) and control diet (CO).

**Comparison**	**Five weeks**			**Eight weeks**		
	**p < 0.1**^1^	**p < 0.05**^1^	**p < 0.01**^1^	**p < 0.1**^1^	**p < 0.05**^1^	**p < 0.01**^1^
	
**WT**						
LSF vs CO	0	0	0	3	3	0
HSF vs CO	3	0	0	0	0	0
**PTEN null**						
LSF vs CO	17	3	0	26	12	1
HSF vs CO	286^2^	80^3^	11	189	72	25
**Interaction**						
PTEN null CO vs WT CO	385	198^4^	8	1542	989^5^	296
PTEN null LSF vs WT CO	660	287	42	2379	1718^5^	822
PTEN null HSF vs WT CO	15	0	0	3380	2390^5^	1204

^1 ^Benjamini and Hochberg adjusted P-values between the two groups.

^2 ^Further details of these transcripts are provided in Figure 8 and Table 2.

^3^Human orthologues of these transcripts were identified and compared to results from a human dietary intervention study performed previously (Figure 9).

^4^Details of these transcripts and their expression are provided in Figure 10A and Additional file 1 - Table S1.

^5^Functional analyses of these transcripts are provided in Table 3, and further analyses in Figure 11.

**Figure 8:**
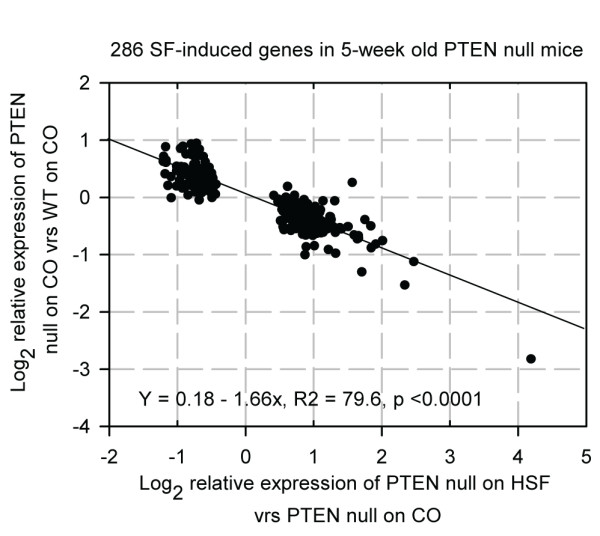
**SF reverses changes in gene expression induced by the PTEN deletion**. Comparative analyses of changes in gene expression in five-week old PTEN null mice fed SF diet compared to PTEN null mice fed control diet, and expression of the same genes in PTEN null mice fed control diet to WT mice fed control diet. The inverse association indicated that SF suppresses gene changes associated with PTEN deletion.

We found very few changes in prostate transcript expression induced by SF in the WT mice at 5 or 8 weeks of age (Table 1). In contrast, there were many dose-dependent changes in gene expression induced by SF in the PTEN null background (Table 1), suggesting that the effects of SF are dependent upon activation of PI3K/AKT signalling pathway due to PTEN deletion. This would explain how SF may have selective effects towards cells that are liable to proliferate. For further insight into the changes induced by the HSF diet, we undertook functional analyses with GenMAPP and found that there was a high frequency of genes specifically associated with PI3K/AKT mediated metabolic processes, such as amino acid, fatty acid, glucose metabolism and apoptosis (Table 2). When we compared the changes in expression of the genes induced by HSF to those that occurred in the PTEN null background compared to the WT background, we found that there was an inverse association. This indicates that the SF was able to suppress pAKT-mediated gene expression (Figure 8).

**Table 2:** Functional analysis using GenMAPP of genes changed in response to the high sulforaphane diet in five week old PTEN null mice compared to the control diet.

	Number Changed*	Number Measured**	Adjusted P-value***
	
Amino acid metabolism			
Mm_Metabolism-of-amino-acids-and-related-nitrogen-containing-molecules_Reactome-COREG	26	225	0.001
Mm_Valine-leucine-and-isoleucine-biosynthesis_KEGG-COREG	11	48	0.001
Mm_Glyoxylate-and-dicarboxylate-metabolism_KEGG-COREG	12	63	0.004
Mm_Glycine-serine-and-threonine-metabolism_KEGG-COREG	14	85	0.004
Mm_Catabolic-Pathways-for-Arginine-Histidine-Glutamate-Glutamine-and-Proline_BioCarta-COREG	12	67	0.004
Mm_Acetylcholine_Synthesis-COREG	11	57	0.004
Mm_Pyruvate-metabolism_KEGG-COREG	18	152	0.008
Mm_Urea-cycle-and-metabolism-of-amino-groups_KEGG-COREG	14	104	0.010
Mm_Urea_cycle_and_metabolism_of_amino_groups_KEGG-COREG	14	115	0.015
Mm_Butanoate-metabolism_KEGG-COREG	19	200	0.019
Mm_Oxidative-decarboxylation-of-pyruvate-and-TCA-cycle_Reactome-MEGINT	13	108	0.019
Mm_Propanoate_metabolism	4	13	0.02
Mm_Arginine-and-proline-metabolism_KEGG-COREG	17	176	0.02
Mm_Catabolic-Pathways-for-Methionine-Isoleucine-Threonine-and-Valine_BioCarta-COREG	5	22	0.026
**Fatty acid metabolism**			
Mm_Fatty_Acid_Beta_Oxidation_3_BiGCaT-COREG	18	69	< 0.001
Mm_Fatty_Acid_Beta_Oxidation_Meta_BiGCaT-COREG	26	174	< 0.001
Mm_Fatty_Acid_Beta_Oxidation_1_BiGCaT-COREG	19	140	0.003
Mm_Fatty_Acid_Beta_Oxidation_3_BiGCaT	4	8	0.004
Mm_Fatty_Acid_Beta_Oxidation_3_BiGCaT-MEGINT	7	24	0.004
Mm_Fatty_Acid_Beta_Oxidation_Meta_BiGCaT	8	32	0.004
Mm_Unsaturated_Fatty_Acid_Beta_Oxidation_BiGCaT	3	6	0.009
Mm_Fatty_Acid_Beta_Oxidation_2_BiGCaT	3	6	0.009
Mm_Fatty_Acid_Synthesis_BiGCaT-COREG	14	103	0.009
Mm_Fatty_Acid_Beta_Oxidation_2_BiGCaT-COREG	8	38	0.009
Mm_Fatty_Acid_Beta_Oxidation_Meta_BiGCaT-MEGINT	11	82	0.020

**Glucose metabolism**			
Mm_Glycolysis_and_Gluconeogenesis-COREG	22	204	0.008
Mm_Glutamate-metabolism_KEGG-COREG	19	165	0.008
Mm_Glycolysis-Gluconeogenesis_KEGG-COREG	20	217	0.019
Mm_Metabolism-of-glucose-other-sugars-and-ethanol_Reactome-COREG	21	248	0.020

**Apoptosis**			
Mm_Opposing-roles-of-AIF-in-Apoptosis-and-Cell-Survival_BioCarta-COREG	12	50	< 0.001
Mm_HSP70_and_Apoptosis-COREG	12	68	0.007
Mm_Role-of-Mitochondria-in-Apoptotic-Signaling_BioCarta-COREG	12	70	0.008
Mm_Apoptosis_Reactome-COREG	12	76	0.009
Mm_Apoptotic-Signaling-in-Response-to-DNA-Damage_BioCarta-COREG	12	87	0.015
Mm_Induction-of-apoptosis-through-DR3-and-DR4-5-Death-Receptors _BioCarta-COREG	11	75	0.015
Mm_Caspase-Cascade-in-Apoptosis_BioCarta-COREG	11	95	0.059

**Krebs-TCA cycle**			
Mm_Krebs-TCA_Cycle	11	29	< 0.001
Mm_Citrate-cycle-TCA-cycle_KEGG-COREG	26	167	< 0.001
Mm_Krebs-TCA_Cycle-COREG	30	216	< 0.001
Mm_Oxidative-decarboxylation-of-pyruvate-and-TCA-cycle_Reactome-COREG	20	124	< 0.001
Mm_Krebs-TCA_Cycle-MEGINT	17	153	0.011

**Other**			
Mm_Electron_Transport_Chain-COREG	42	298	< 0.001
Mm_Shuttle-for-transfer-of-acetyl-groups-from-mitochondria-to-the-cytosol_BioCarta-COREG	18	100	< 0.001
Mm_Ceramide-Signaling-Pathway_BioCarta-COREG	13	77	0.004
Mm_Reductive-carboxylate-cycle-CO2-fixation-_KEGG-COREG	13	74	0.004
Mm_Malate-aspartate-shuttle_BioCarta-COREG	9	41	0.007
Mm_Nitrogen-metabolism_KEGG-COREG	13	81	0.008
Mm_Ribosomal_Proteins	11	63	0.008
Mm_Electron_Transport_Chain	11	66	0.009
Mm_Stress-Induction-of-HSP-Regulation_BioCarta-COREG	11	67	0.009
Mm_D4-GDI-Signaling-Pathway_BioCarta-COREG	11	71	0.011
Mm_Carbon-fixation_KEGG-COREG	19	187	0.015
Mm_Nucleotide-metabolism_Reactome-COREG	34	517	0.019

Only pathways with adjusted P-values ≤ 0.05 are shown.

*Refers to the transcripts from the input 286 transcript list (adjusted P ≤ 0.1) that are present in the specific pathway. **Refers to the total number of transcripts that are present in the pathway.***P-values were calculated in GenMAPP using a non-parametric statistic based on 2000 permutations of the data and further adjusted for multiple testing by Westfall-Young adjustment.

### A comparison between gene expression modulated by SF in mice and gene expression modulated by a broccoli-rich diet in humans

**Figure 9:**
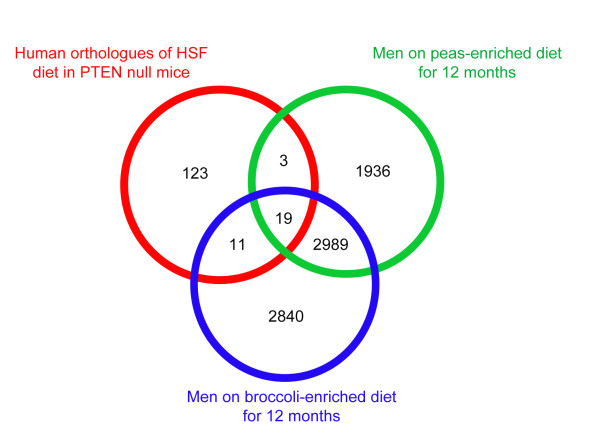
**Comparison between gene changes in men on a dietary intervention and those in mice fed SF**. Overlap between human genes that are altered in expression after 12 months by a diet enriched in either peas or broccoli in men, with human orthologues of genes changed in expression by SF in a mouse PTEN null background at five weeks. If all gene changes were independent from each other, 1.5 genes would have been expected to be in common to all the three data sets as opposed to 19.

One rationale of feeding SF to PTEN knock out mice is that it is a model for the potential protective effects of broccoli on the development of prostate cancer in humans, partially validated by our observations that PTEN deletion may occur early in human prostate carcinogenesis (Figure 1). We thus sought to see if there was a significant overlap in the genes induced by SF in the PTEN null mice with previously obtained data on the changes in gene expression in sequential prostate biopsies of men who were deemed to be at risk of prostate cancer following a 12 month dietary intervention with either broccoli or peas, as previously described [13]. In this study, men who had previously received a diagnosis of HGPIN, and exhibited heterogeneity in expression of PTEN and pAKT (Figures 1 and 2), were randomized into two dietary intervention groups, in which they were required to consume either 400 g of peas per week or 400 g of high glucosinolate broccoli per week for 12 months but to otherwise follow their normal diet which was quantified through the use of diet diaries. Global gene expression was quantified through the use of human Affymetrix arrays in sequential biopsies obtained before and after the intervention from each of the volunteers, as previously described. We converted mouse genes that changed in expression following SF intervention into their human orthologues using the HomoloGene database at the NCBI website. We initially undertook comparisons between the human 156 Affymetrix probe sets orthologues of the 80 mouse genes that were altered in expression by HSF after five weeks (Table 1) with human genes that were altered in expression in prostate biopsy samples taken from volunteers before and after a 12-month intervention with the broccoli-enriched diet (5859 Affymetrix probesets). We found that 30 probesets were common to both lists, which was significantly greater (p < 0.002) than the 16 probesets that we would have expected by chance. When we compared the changes in gene expression in a similar group of men whose diet had been enriched with peas (4947 Affymetrix probesets), we found that 22 probesets were common to both lists, which was also significantly greater than that which we would expect by chance (14 probesets, p = 0.023). When we combined the three data sets (Figure 9), we found that 19 probesets were common to the three data sets. When we repeated the analysis with the 72 mouse genes that were altered in expression after eight weeks, we found no significant overlaps with the human data.

### Interaction of PTEN deletion and SF in modulating gene expression

We then compared gene expression profiles between WT mice fed the control diet and PTEN null mice that had been fed either the control diet or diets supplemented with SF to investigate the combined effects of PTEN deletion and diet. When tissue was analysed at five weeks of age, it was apparent that the addition of SF to the diet had two effects. The LSF diet reduced the number of PTEN signature gene changes from 198 to 66, while inducing changes in expression of 221 additional genes that had not previously been perturbed by the PTEN deletion. These additional gene changes induced by LSF were associated with phosphate transport, cell adhesion and extracellular matrix organisation (Additional file 2 - Table S2). The HSF diet suppressed all changes in gene expression that had previously been induced by the PTEN deletion, and did not induce any additional changes in gene expression (Table 1).

**Figure 10:**
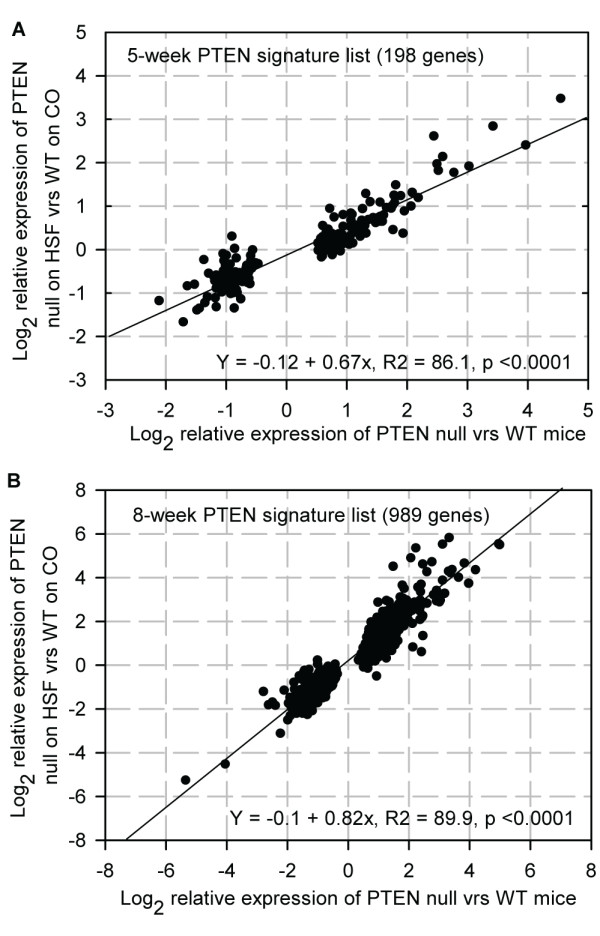
**Effect of HSF on the expression of the PTEN null signature genes**. **A**, Expression of the five week 198 PTEN signature list in PTEN null mice fed the control diet compared to WT mice fed control diet, and 5-week old PTEN null mice supplemented with HSF diet compared to WT mice fed control diet. The addition of SF results in the magnitude of change in expression of each gene being suppressed by on average 33% (see Table 1 and Additional file 1 - Table S1). **B**, Expression of the eight week 989 PTEN signature list in PTEN null mice fed control diet compared to WT mice fed control diet, and 8-week old PTEN null mice supplemented with HSF diet compared to WT mice fed control diet (see Table 1).The addition of SF results in the magnitude of change in expression of each gene being suppressed by on average 18%.

The lack of statistically significant differences in gene expression between WT mice and PTEN null mice fed the HSF diet appeared inconsistent with epithelial hyperplasia observed in these mice at five weeks of age. We therefore compared the extent of the magnitude of changes in expression of the PTEN signature genes induced by the PTEN deletion with the control diet to that when the diet was supplemented with SF. We found that the HSF diet had not entirely suppressed changes in the PTEN signature genes, but attenuated the extent of the changes by about 33% (Figure 10A, Additional file 1 - Table S1), so that none of them alone reach statistical significance. Likewise, the LSF suppressed the extent of changes in the PTEN signature genes by 23% (Additional file 1 - Table S1). As there was similar suppression in all the PTEN signature genes, regardless of whether they had been up- or down-regulated or their ontological association, it is likely that SF has a direct effect on suppressing PI3K/AKT signalling. However, in this model the extent of suppression of PTEN null-mediated gene expression was insufficient to suppress pathological changes in prostate tissue.

**Figure 11:**
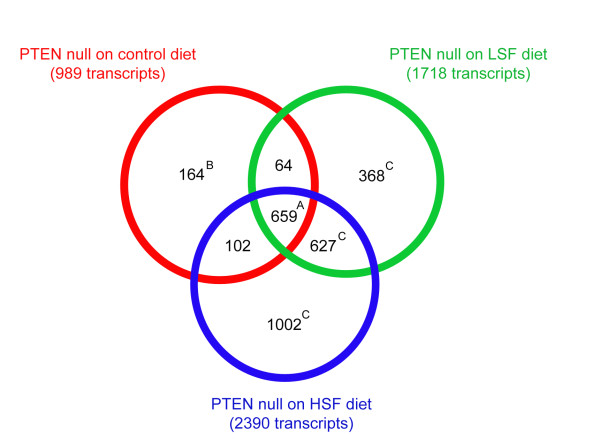
**Comparison of gene changes in PTEN null mice on different diets compared to WT mice fed the control diet**. Overlap of differentially expressed transcripts (P ≤ 0.05) of 8-week old PTEN null mice on the control diet (989 transcripts, 'PTEN signature transcripts', Table 1), low sulforaphane (LSF, 1718 transcripts, Table 1) and high sulforaphane (HSF, 2390 transcripts, Table 1) diet each compared to 8-week old WT mice on the control diet. **A**, Functional analysis by GenMAPP of these genes (data not shown) confirmed that they are enriched for genes involved in immune response, inflammatory response and response to wounding, two of which are common pathways between all three diets (Table 3). **B**, Details of these genes are shown in Additional file 3 - Table S3. **C**, Functional analysis of these genes is shown in Additional file 4 - Table S4.

When gene expression profiles were analysed in prostate tissue from eight week old mice, SF could again be observed to have two effects. First, the LSF and HSF diets reduced the number of PTEN signature gene changes from 989 to 723 and 761, respectively (Figure 11). This more modest reduction was also reflected in the similarity of changes in the magnitude of expression of the majority of the 989 genes in PTEN null mice fed either the control diet, or diets supplemented with LSF or HSF (Figure 10B). However, both SF diets attenuated the expression of a subset of the PTEN signature genes involved in positive regulation of cell differentiation, cell division and post-translational protein modification (Additional file 3 - Table S3). Secondly, the addition of SF to the diet of PTEN null mice resulted in changes in the expression of additional genes (Figure 11, Additional file 4 - Table S4). Pathway analyses of the genes that were altered in expression due to the PTEN deletion and its interaction with SF is summarised in Table 3. As would be expected, certain pathways, associated with changes induced by the PTEN deletion were common to PTEN null prostate tissue regardless of diet, such as immune and inflammatory response, whereas mice that had diets supplemented with SF had additional changes in pathways associated with cell cycle regulation and apoptosis (Table 3). The change in expression of genes within these pathways were indicative of SF inducing cell cycle arrest and apoptosis (Additional file 5 - Table S5; Additional file 6 - Table S6). For example, the up regulation of cyclin B1 and the down regulation of cyclin D2 are consistent with cell cycle arrest, and the two fold increase in expression of caspases 3 and 7 are consistent with the induction of caspase-induced apoptosis. It is notable that we did not observe significant up regulation of Nrf2 regulated genes.

**Table 3:** Functional analysis using GenMAPP of eight week old PTEN null mice on different diets compared to WT mice on control diet

MAPP Name	Number Changed*	Number Measured**	Adjusted P-value***
	
Control diet			
immune response	40	160	< 0.001
inflammatory response	22	85	< 0.001
Mm_Inhibition-of-Matrix-Metalloproteinases_BioCarta-COREG	6	13	0.025
Mm_mRNA_processing_binding_Reactome-COREG	46	1309	0.022
**Low SF diet**			
immune response	57	160	< 0.001
inflammatory response	36	85	< 0.001
Mm_Bile-acid-biosynthesis_KEGG-COREG	30	105	0.013
Mm_Carbon-fixation_KEGG-COREG	47	187	0.004
Mm_Electron_Transport_Chain-COREG	73	298	< 0.001
Mm_HSP70_and_Apoptosis-COREG	21	68	0.028
Mm_Metabolism-of-glucose-other-sugars-and-ethanol_Reactome-COREG	58	248	0.005
Mm_Monocyte-and-its-Surface-Molecules_BioCarta-MEGINT	9	19	0.028
Mm_Propanoate-metabolism_KEGG-COREG	23	77	0.027
Mm_Ribosomal_Proteins	29	63	< 0.001
response to chemical substance	26	93	0.029
response to wounding	42	152	0.001

**High SF diet**			
immune response	63	160	< 0.001
inflammatory response	40	85	< 0.001
intracellular signaling cascade	64	229	0.024
Mm_Adipogenesis	39	122	0.026
Mm_Adipogenesis-MEGINT.mapp-.mapp-.mapp-.mapp-.mapp-.mapp-	62	208	0.004
Mm_Apoptotic-DNA-fragmentation-and-tissue-homeostasis_BioCarta-COREG	25	66	0.024
Mm_ATM-Signaling-Pathway_BioCarta-COREG	30	77	0.003
Mm_B_Cell_Receptor_NetPath_12	48	137	0.001
Mm_cdc25-and-chk1-Regulatory-Pathway-in-response-to-DNA-damage_BioCarta-COREG	39	121	0.024
Mm_Cell_Cycle_KEGG	29	77	0.010
Mm_Cell-Cycle-Checkpoints_Reactome-COREG	54	157	< 0.001
Mm_Cyclin-E-Destruction-Pathway_BioCarta-COREG	23	53	0.003
Mm_E2F1-Destruction-Pathway_BioCarta-COREG	31	90	0.027
Mm_GPCRDB_Other	3	139	0.025
Mm_Hedgehog_Netpath_10-COREG	46	141	0.004
Mm_IL-3_NetPath_15	30	87	0.030
Mm_IL-5_NetPath_17	26	62	0.003
Mm_Influence-of-Ras-and-Rho-proteins-on-G1-to-S-Transition_BioCarta-COREG	27	72	0.018
Mm_NF-kB-Signaling-Pathway_BioCarta-MEGINT	16	34	0.018
Mm_RB-Tumor-Suppressor-Checkpoint-Signaling-in-response-to-DNA-damage_BioCarta-COREG	40	124	0.019
Mm_Ribosomal_Proteins	25	63	0.015
Mm_Role-of-BRCA1-BRCA2-and-ATR-in-Cancer-Susceptibility_BioCarta-COREG	30	79	0.004
response to wounding	49	152	0.003

Only pathways with adjusted P-values ≤ 0.05 are shown. Pathway analysis was performed on transcript lists that were statistically significant (Benjamini and Hochberg adjusted *P *≤ 0.05,) between the two groups. No fold cutoff was used.

* Refers to the transcripts from the input transcript list identified for each diet that are present in the specific pathway. **Refers to the total number of transcripts that are present in the pathway. ***P-values were calculated in GenMAPP using a non-parametric statistic based on 2000 permutations of the data and further adjusted for multiple testing by Westfall-Young adjustment.

As an overview, the data on changes in gene expression suggest that SF has two effects: it can both suppress PTEN deletion - mediated gene transcription, such as that observed at five weeks, particularly by the HSF diet, as well as inducing additional changes in gene expression. The balance between these two effects, and thus the overall changes in gene expression, depends upon the age of the mouse and the concentration of SF.

### Interaction of PTEN deletion and SF on modulating alternative splicing

The development of high density exon arrays facilitates the global analyses of alternative splicing events. Several statistical approaches have been developed to analyse the large and complex data sets arising from the use of these arrays [16], of which we used FIRMA ('Finding Isoforms using Robust Multichip Analysis') [17], specifically developed for analysing Affymetrix exon array data and implemented in the *aroma.affymetrix *package of R/Bioconductor [18]. Briefly, FIRMA scores each exon as to whether its probes deviate from the expected transcript expression level and are used to perform further statistics to identify alternative splicing events.

Initially, we compared changes in exon FIRMA profiles induced by SF in WT or PTEN null genetic backgrounds. In a similar manner to that observed for transcriptional changes, SF induced only potential splicing events in the PTEN null background implying the need for activated PI3K/AKT signalling (Table 4). However, the greatest number of exon skipping/inclusion was evident when comparisons were made between WT mice and PTEN null mice whose diets had been supplemented with SF (Table 4, Additional file 7 - Table S7), indicating that SF interacts with the PTEN deletion to induce alternative splicing. The functional pathways of the genes that were potentially alternatively spliced by SF are shown in Additional file 8 (Table S8). A large proportion of genes for which there was indication of alternative splicing also showed perturbed overall transcript expression by SF (74% for HSF and 47% for LSF), suggesting that alternative splicing by SF is coupled to transcription.

**Table 4:** Exons that are significantly changed in PTEN null and WT mice following SF-rich diets (LSF, HSF) and control diet (CO).

Comparison	Five weeks			Eight weeks		
	p < 0.1^1^	p < 0.05^1^	p < 0.01^1^	p < 0.1^1^	p < 0.05^1^	p < 0.01^1^
**WT**						
LSF vs CO	0	0	0	0	0	0
HSF vs CO	3	0	0	0	0	0
**PTEN null**						
LSF vs CO	18	6	1	106	31	0
HSF vs CO	318	37	2	94	24	3
**Interaction**						
PTEN null CO vs WT CO	2	0	0	36	2^2^	0
PTEN null LSF vs WT CO	51	16	1	602	287^2^	28
PTEN null HSF vs WT CO	4	1	1	872	267^2^	35

^1 ^Benjamini and Hochberg adjusted P-values between the two groups.

^2 ^Details of these exons are provided in Additional file 7 - Table S7.

**Figure 12:**
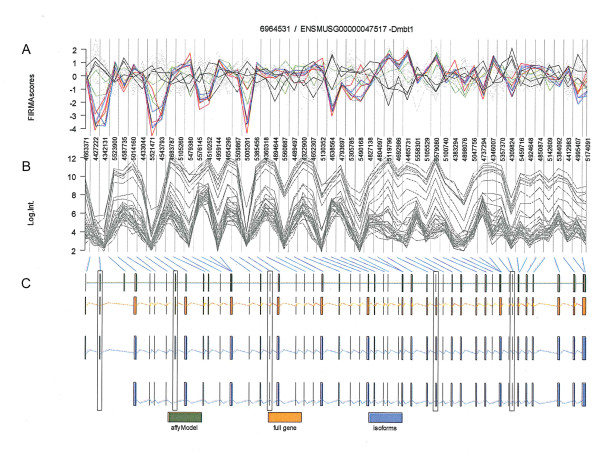
**Exon array analyses of alternative splicing of Dmbt1 gene with FIRMA**. **A**, FIRMA scores calculated from array data for all the mice are plotted in the top graphic. Each line represents values from a single mouse. Highlighted in colour are 8-week old mice [black, WT on control diet; green, PTEN null on control diet; blue, PTEN null on LSF diet; red, PTEN null on HSF diet]. **B**, The normalised logged intensities of each probeset are plotted (note that certain exons are represented by more than one probeset). The blue connecting lines map these probesets to (**C**) gene models as defined by Affymetrix (green) and Ensembl (orange). Transcript isoforms known for this gene are plotted in dark blue. The longer isoform is designated in Ensembl as Dmbt1-202 and the shorter as Dmbt1-201 (see Figure 13). The five regions highlighted in the plot show exons with statistically significant difference in FIRMA scores between 8-week PTEN null mice on HSF diet and WT mice on control diet.

**Figure 13:**
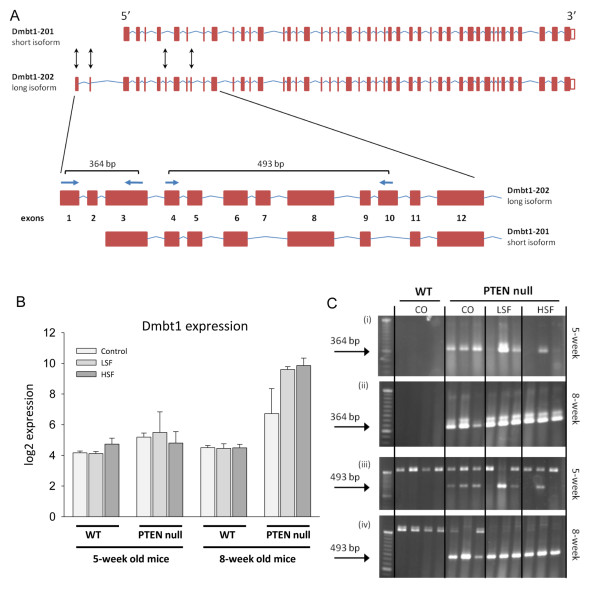
**RT-PCR analyses of alternative splicing of Dmbt1**. There are two known transcript isoforms for this gene: the short isoform designated in Ensembl as Dmbt1-201 and the long isoform Dmbt1-202, which has four additional exons (shown with the black arrows). **A**, Two sets of primers were designed on either side of exons 2 and 7 that were shown to be alternatively spliced in response to the high SF diet. Primer location and expected products are shown. Both sets of primers were designed to give a defined product from the long Dmbt1 isoform but no product from the short isoform. **B**, Gene expression of Dmbt1. After eight weeks, there was a significant effect on expression of the DMBT1 transcript by PTEN genotype (p > 0.001), SF (p = 0.008), and by a PTENxSF interaction (p = 0.007). **C**, Amplified products from five-week old (i, iii) and eight-week old (ii, iv) mice show the effect of diet on the Dmbt1 loci flanking exon 2 (i, ii) and exon 7 (iii, iv). Note that during amplification of the locus flanking exon 7 there is a high MW product that corresponds to the presence of genomic DNA. However, the 493 bp mRNA product corresponding to the long Dmbt1 isoform is preferentially amplified.

We selected DMBT1 (Deleted in Malignant Brain Tumors 1), a gene involved in innate immunity, cell differentiation and tumour suppression [19], to explore further the effects of PTEN genotype and SF on gene expression and splicing. FIRMA analyses suggested significant effects of PTEN deletion and SF on splicing of several exons (Figure 12) that was consistent with two known splice variants of DMBT1, the shorter isoform, designated as DMBT1-201 which is missing exons 1, 2 and 7 and 10, and the longer isoform, DMBT1-202 which contains these exons (Figure 13A). We designed PCR primers within exons 1, 3, 4 and 10 which would distinguish the two isofoms (Figure 13A).

At five weeks of age, neither PTEN nor SF had an effect on overall expression of the DMBT1 transcript (Figure 13B). However, we found that all WT mice, regardless of diet, lacked amplification of PCR products due to the likely absence of exons 1 and 10, consistent with the expression of the shorter isoform. In contrast, all PTEN null mice fed the control diet had expression of the longer isoform (Figure 13C(i) and (iii)). However, five-week old PTEN null mice fed the SF diets varied in which isoform they expressed (Figure 13C(i) and (iii)), indicative of a possible effect of SF on suppression of alternative splicing induced by PTEN deletion, similar to the manner by which SF can suppress PTEN deletion-mediated gene expression at five weeks (Figures 8 and 10A, Additional file 1 - Table S1).

After eight weeks, there was a significant effect on expression of the DMBT1 transcript by PTEN genotype (p > 0.001), SF (p = 0.008), and by a PTENxSF interaction (p = 0.007, Figure 13B), illustrating the manner by which PTEN and diet can interact to determine gene expression, evident from Table 1. Eight-week old WT mice, regardless of diet, express the short isoform whereas PTEN null mice, again regardless of diet, all expressed the long isoform (Figure 13C(ii) and (iv)). It is not possible to say from these preliminary analyses whether at eight weeks the enhanced transcription of DMBT1 by SF induces the PTEN-mediated alternative splicing. However, the large number of genes within PTEN null mice on SF diets that express alternatively spliced transcripts compared to WT mice on the control diet (Table 4, Additional file 7 - Table S7) suggests that this may be the case. Further studies of the interaction between PTEN genotype and diet are required.

## Discussion

Studies on the effects of dietary components, such as isothiocyanates and fish omega-3 fatty acids, on cancer progression have focused on reporting their effects on tumour growth and development [20-22]. Likewise studies on gene expression associated with carcinogenesis in animal models have frequently only centred on changes associated with the later stages of tumour growth and progression to metastasis [14]. In this study we are concerned with changes in gene and protein expression that occur in the very early stages of prostate carcinogenesis, prior to tumour development and how they may be modulated by diet. Up to now preliminary results indicate that green tea catechins are promising in suppressing cancer progression in individuals with HG-PIN but a mechanistic explanation for this effect is not yet clear [23]. In the present study, we sought to explore the possibility that the dietary isothiocyanate sulforaphane has contrasting effects on cells and tissues with differential PTEN expression. This may provide a partial explanation of the inverse association between diets rich in broccoli and the risk of prostate cancer [12,24], and also suggest potential therapeutic applications of sulforaphane and related compounds.

Initially, we found that in men that are at risk of prostate cancer following a previous diagnosis of HGPIN, there is considerable variation in PTEN and pAKT expression that is not associated with obvious histological abnormalities. Heterogeneity of expression would be consistent with the characteristic emergence of several cancer foci in the human prostate. PTEN also varied in its localisation of expression. While intense pAKT expression was associated with lack of both nuclear and cytoplasmic PTEN expression, we also observed loss of nuclear expression of PTEN without activation of pAKT. While the function of cytoplasmic PTEN is to counteract increased levels of PIP3 by dephosphorylating to PIP2, thereby preventing activation of AKT and downstream signaling, the function of nuclear PTEN is not as well defined but may be independent of its phosphatase activity [25]. Nuclear PTEN function leads to p53-mediated G(1) growth arrest, cell death, and reduction of reactive oxygen species production [26]. Recently, nuclear PTEN has also been linked in controlling chromosomal integrity by acting on chromatin to regulate expression of Rad51, which reduces the incidence of spontaneous DNA double-strand breaks [27].

To determine whether PTEN and subsequent perturbation of the PI3K/AKT signaling pathway would play a significant role in sulforaphane-mediated effects on prostate carcinogenesis, we initially studied the human prostate cancer cell line PC3, which has a PTEN deletion, and found that it is more sensitive to growth inhibition by SF than the PNT1A cell line that has wild type PTEN expression. At low concentrations, SF enhanced the growth of PNT1A, similar to that previously reported for non-cancerous prostate primary epithelial cells [7]. However, as PC3 and PNT1A cell lines would have other differences in their genetic background, we sought to investigate the biological activity of SF in the mouse prostate-specific PTEN deletion model, where progression to carcinogenesis parallels that of human disease. By comparing PTEN-deficient and WT littermates, which differ only in the expression of Cre recombinase and PTEN, we unambiguously showed that SF has much greater activity in a PTEN-deficient background, indicating selectivity towards cells that are at risk of carcinogenesis or tumour tissue itself. In addition, these effects are occurring at the early initiating stages of prostate carcinogenesis before malignant transformation has occurred.

The effects of SF on gene expression in PTEN-deficient prostate tissue were complex. After five weeks, the main effect is to ameliorate PTEN null-mediated gene expression, so that SF itself acted as a surrogate PTEN tumour suppressor, although its effects were not sufficient in this model to reverse the histopathological changes induced by the knock out of PTEN in all prostate epithelial cells. At a later stage, while SF continued to inhibit a subset of PTEN null-mediated gene expression, it also changed the expression of other genes that are associated with cell cycle arrest and apoptosis. For example, the HSF diet up-regulated cyclins A2, B1, B2, E1 and E2, down-regulated cyclin D2 and induced their associated cyclin-dependent kinases, Cdk2 and 6 (Additional file 5 - Table S5). These results are consistent with previous studies. Cell cycle regulation by SF has been reported in prostate and colon cell lines in which G1 phase cell cycle arrest was associated with protein down-regulation of cyclin D1 [28,29], and in non-transformed T lymphocytes where SF reduced cyclin D2 [30]. Gene expression of cyclin D2 was also down-regulated in small intestinal polyps of ApcMin/+ mice after 3 days of SF treatment[31]. Increased protein expression of cyclin B1 has been reported to be induced by SF in human colon and breast cells [32,33]. SF also resulted in a greater than 2-fold increase in expression of caspases 3 and 7 (Additional file 6 - Table S6), consistent with induction of apoptosis in a caspase-dependent manner previously observed in a variety of cell and animal models by SF [6]. For example, SF increased caspase-3 activity in cultured PC3 human prostate cancer cells [5,34] and broccoli sprouts - a rich source of SF - retarded tumour growth associated with caspase-3 cleavage in TRAMP mice, an alternative model of human prostate cancer [3].

Isothiocyanates such as SF are known activators of the NF-E2-related factor 2 (NRF2) transcription factor-signaling pathway [6], and previous studies have reported enhanced expression of NRF2-regulated genes in small intestine and liver of wild type mice [35,36]. Surprisingly, we did not find any enhanced expression of NRF2-regulated genes in either wild type or PTEN null prostate tissue. This may be due to the exposure of prostate tissue in our mouse model to SF being significantly lower than that used in cell studies and other mouse studies, in order to better reflect routine dietary exposure, combined with the probable greater sensitivities of liver and small intestine compared to prostate to any given dose of SF due to topological exposure and first pass effects, respectively. However, it is noteworthy that a previous study also reported lack of enhanced expression of NRF2-regulated genes through dietary intervention with SF in intestinal polyps of APCMin/+ mice [31], suggesting tissue specific effects. Moreover, an acute intervention with standard broccoli and a 12-month dietary intervention with a broccoli-rich diet did not induce NRF2-regulated genes in gastric mucosa [37] and prostate tissue [13], respectively, in human volunteers.

In this study, using the prostate-specific PTEN deletion mouse model we have provided evidence that in a PTEN null background, addition of SF to the diet can ameliorate the effects of the PTEN deletion. It is, therefore, of considerable interest to know whether dietary intervention in men who are at risk of prostate cancer, partly through PTEN deletion or activation of PI3K/AKT signaling, may have a similar effect. To test this we compared changes in gene expression induced by SF in PTEN-null mice with previously reported changes in human prostate tissue following diets enriched in either broccoli (a dietary source of SF) or peas. We unexpectedly found evidence that both diets may induce similar changes, although the overlap with the broccoli diet was of greater statistical significance. It is conceivable, then, that in addition to SF derived from broccoli, some dietary component common to both diets may be able to suppress the downstream effects of PTEN deletion. Possible factors may be lignans, such as secoisolariciresinol, and flavonoids which are present in both peas and broccoli, and which have been associated with reduction in prostate cancer risk [38,39]. Lignans are converted by intestinal microflora to enterodiol and enterolactone, which have been shown to suppress pAKT expression in cell models [40] and certain flavonoids have been shown to modulate PI3K/AKT signalling [41].

The development of exon arrays has facilitated global analysis of alternative gene splicing, and enables more precise interpretation of microarray data. Alternative splicing of primary RNA transcripts may have a variety of functions, including the expression of different protein isoforms with different functional properties and the regulation of gene expression through nonsense-mediated decay [42]. Serine-rich (SR) proteins function as important regulators of alternative mRNA splicing; kinases and protein phosphatase 1 control the reversible phosphorylation status of these proteins that determines splice site selection and cellular location [43,44]. pAKT has been shown to phosphorylate the SR proteins SRp40, SF2/ASF and 9G8, which determine alternative spicing of PKC and fibronectin mRNA [45,46]. Furthermore, insulin has been shown to induce the alternative splicing of PKC via PI3K/AKT activation [47]. Thus, it may be expected that hyperactivation of PI3K/AKT signaling through PTEN deletion may lead to alternative splicing of genes, which may have important biological consequences. While exon arrays have been used to compare tissue- and tumour-specific splicing in human tissues [48,49], we are unaware of any reported analyses that have specifically focussed on PTEN deletions or assessed the role of diet in inducing alternative splicing.

We provide evidence that the interaction between PTEN deletion and supplementation of diet with SF can result in the alternative splicing of many genes. This is likely to be a complex interaction of both PTEN deletion and SF affecting phosphatase activity, as has been shown for other isothiocyanates [50] and overall changes in gene transcription induced by SF. Indeed, the HSF diet altered expression of two regulatory subunits of protein phosphatase 1 that may partly explain the observed effects. An example of perturbed alternative splicing is the DMBT1 gene where PTEN deletion promoted the expression of the longer isoform and SF affected this process. The human DMBT1 gene, involved in terminal differentiation of epithelial cells, is located on chromosome 10 q, a region often deleted in prostate cancer and also containing the PTEN tumour suppressor [51]. It is also important to note that in a previous study bioinformatic analyses of the changes in gene expression in men with a broccoli-rich diet reported changes in mRNA processing, indicative of alternative splicing [13]. However, as this previous study did not use exon arrays, it is not yet possible to quantify the effects of diet on splicing in human prostate tissue.

## Conclusion

Suppression of PI3K/AKT signaling has become a major potential target of therapeutic or dietary intervention to prevent the progression of prostate cancer, and cancer at other sites [52]. Tumours with PTEN loss and enhanced PI3K/AKT activity have been shown to be less susceptible to calorific restriction than other tumours [53], but are restricted in growth through a diet rich in omega-3 polyunsaturated fatty acids [20]. In our current study, we focus on the manner by which SF perturbs gene expression in non-malignant PTEN null tissue, as opposed to later stages of tumourigenesis and metastasis. Further studies are required to assess the effect of SF on PTEN null-mediated prostate tumourigenesis, although probably with a less aggressive model more akin to the temporal development of human prostate cancer [54,55]. Here, we clearly demonstrate that SF interacts with PTEN-deficient cells and tissues to modulate gene expression and alternative splicing events. This may explain how diets rich in broccoli, the dietary source of SF, can reduce the risk of incidence of prostate cancer and the progression of localised prostate cancer to more aggressive forms of this disease [12]. Furthermore, it emphasises the complexities of the interaction between genotype, gene expression and diet.

## Materials and methods

### Ethics statement

This study was conducted according to the principles expressed in the Declaration of Helsinki. The study was approved by the East Norfolk & Waveney Research Governance Committee and the Norfolk Research Ethics Committee (references 05/Q0101/9 and 09/H0311/96). All patients provided written informed consent for the collection of samples and subsequent analyses.

All animals were handled in strict accordance with good animal practice and all animal work was approved by the University of East Anglia Animal Ethics Committees and covered by the appropriate licences under the UK Home Office Animal Procedures Act, 1986 (PPL 80/1799).

### Human subjects

Trans-Rectal Ultra Sound (TRUS)-guided needle biopsy tissues were obtained from men who had previously received a diagnosis of high grade prostatic intraepithelial neoplasia (HGPIN) via the Urology Clinic at the Norfolk and Norwich University Hospital as previously described [13]. Briefly, RNA was extracted from whole biopsy tissues for gene expression analysis and adjacent biopsies were evaluated by histology. Although the latter did not contain neoplastic cells, we cannot exclude the presence of these cells in the biopsies used for gene expression analysis. Details of the volunteers (age, BMI, PSA, GSTM1 genotype) are as previously described [13].

### Cell culture and proliferation assays

The human post-pubertal prostate normal (PNT1A) cell line and the human Caucasian prostate adenocarcinoma (PC3) cell line were obtained from the European Collection of Cell Cultures (ECACC). PNT1A and PC3 cells were routinely cultured as monolayers in RPMI-1640 and HAMS media, respectively, supplemented with 10% fetal bovine serum (FBS) in a humidified atmosphere containing 5% CO_2 _at 37°C. Cells were grown to 70-80% confluence before incubation in complete media with various concentrations of SF. Cell viability was determined following a 24 h treatment with either SF or vehicle DMSO using the WST-1 Cell Proliferation Reagent (Roche Applied Science). Cell proliferation was assessed by ELISA following BrdU incorporation (Roche Applied Science).

### Animal Husbandry and Genotyping

PTEN^L/L^;C^+ ^mice were generated by crossing ARR2Probasin-Cre transgenic mice, PB-Cre4 [56], to PTEN^L/L ^mice [57] as described before [14]. Only F2 generation male offspring (PTEN^L/L^;C^+ ^) and their littermate controls (PTEN^L/L^;C^-^) were used in this study. For simplicity PTEN^L/L^;C^+ ^mice will be referred to as PTEN null and PTEN^L/L^;C^- ^mice as wild type (WT). PB-Cre4 mice (strain B6.D2-Tg(Pbsn-cre)4Prb) were obtained from the Mouse Models of Human Cancers Consortium (MMHCC) of the National Cancer Institute, US, and PTEN^L/L ^mice (strain C;129S4-PTEN*^tm1Hwu^*/J) from the Jackson Laboratories (http://jaxmice.jax.org). Mice were housed in a room with controlled humidity, temperature and light within the Disease Modelling Unit at the University of East Anglia.

Genotyping for the Cre recombinase and PTEN genes was performed by PCR using tail tip DNA. DNA was extracted using the QIAGEN DNA blood and tissue kit (QIAGEN). For the PB-Cre4 genotyping we used two sets of primers, one specific to the transgene producing a 199 bp product (5'-ACC AGC CAG CTA TCA ACT CG-3' and 5'-TTA CAT TGG TCC AGC CAC C-3') and the other as an internal control producing a 324 bp product (5'-CTA GGC CAC AGA ATT GAA AGA TCT-3' and 5'-GTA GGT GGA AAT TCT AGC ATC ATC C-3'). Excision of exon 5 of the PTEN gene was determined by the size of the product from the PCR amplification using the primers 5'-ACT CAA GGC AGG GAT GAG C-3' and 5'- GCC CCG ATG CAA TAA ATA TG-3', where 1.2 Kb results from a wild-type PTEN allele and 1.3 kb from a mutant allele.

### Diet formulations and experimental design

Animal feed consisted of basal diet (AIN-93G, control diet) or basal diet enriched with either 0.1 μMol SF/g diet (LSF diet) or 1 μMol SF/g diet (HSF diet). D, L-SF was purchased from LKT laboratories (St. Paul, MN, USA) and was incorporated into the basal diet by Harlan Teklad (Madison, WI, USA). SF levels were confirmed in the diets by LC-MS as described previously [58]. It is noteworthy that that we chose lower SF levels compared to previous studies, ranging between 1.2-3.4 μMol SF/g [4,21], so that the intake would reflect more physiological dietary exposure. Homozygous male PTEN null mice and their littermate controls were randomly assigned to the experimental and control diets, which they received from weaning *ad libitum *for a duration of either two weeks or five weeks. Animals were sacrificed by CO_2 _asphyxiation and cervical dislocation at five and eight weeks of age. At the time of sacrifice, the total mouse weight was recorded. Mouse prostates were removed immediately and either placed in RNAlater^® ^and stored at -20°C for subsequent expression analysis (n = 3 per group) or fixed in 10% formalin, embedded in paraffin wax, and used for histopathological evaluations and immunohistochemichal (IHC) staining (n = 3-5 per group).

### Histopathology and immunohistochemistry

Dissected tissue samples from mouse and biopsy samples from human volunteers were fixed in 10% formal-saline, embedded in paraffin wax and sections approximately 4 μm thick were cut. One slide from each tissue sample was stained with hematoxylin and eosin (H&E). For immunohistochemical assessment of both the mouse and the human tissue samples, the slides were deparaffinised in three changes of xylene and rehydrated through graded ethanols (100% to 50%). Heat-induced antigen retrieval was performed using citrate buffer pH 6.0 (Dako UK Ltd., Ely, Cambridgeshire, UK). Endogenous peroxidises were quenched using 3% hydrogen peroxide in PBS or TBS and then blocked with 1%BSA in PBST or 5% normal goat serum in TBST appropriate to manufacturer's instructions for each primary antibody. Primary antibodies were diluted in appropriate buffer according to the supplier's instructions: Anti-mouse Ki67 rat monoclonal (Dako, #M7249) diluted 1:50 in PBST; Anti-PTEN(D4.3) rabbit monoclonal diluted 1:125 in SignalStain^® ^Antibody Diluent(Cell Signaling, New England Biolabs, Hitchin, UK); Anti-pAKT (Ser473) (D9E) rabbit monoclonal diluted 1:50 in SignalStain^®^; Anti-Phospho-mTOR (Ser2448)(49F9) rabbit monoclonal diluted 1:50 in TBST; (Cell Signaling antibodies: #9188; #4060 and #2976 respectively). All were incubated at 37°C for 30 mins. Detection was performed using the appropriate secondary antibody with Vector ABC Elite kits and DAB substrate kit (Vector Laboratories, Peterborough, UK).

All slides were counterstained with Mayer's haematoxylin (Surgipath Europe Ltd, Peterborough, UK), dehydrated through graded ethanols (50% to 100%), and mounted with cover slips. Slides were visualised using an Olympus BX60 (Olympus, Japan) microscope with ProgRes^® ^Capture Pro 2.1 software (Jenoptik, Germany). Histopathological assessment was performed according to the classification of the Bar Harbor Meeting of the Mouse Models of Human Cancer Consortium Prostate Pathology Committee [59]. Stained sections were evaluated by two independent researchers. The immunohistochemical quickscore (Q) was determined for each by multiplying the estimated percentage of positively stained cells (P) by the intensity of staining (I). Estimates of intensity were scored as follows: 1 for weak staining; 2 for moderate staining and 3 for strong staining (Q = P × I; maximum 300).

### Gene expression analysis

RNA from mouse prostate tissue stored in RNAlater^® ^was isolated using RNeasy Mini kit (QIAGEN) and gene expression profiling was performed using the Affymetrix GeneChip^® ^Mouse Exon 1.0 ST Array (Affymetrix, Santa Clara, CA) at the Nottingham Arabidopsis Stock Centre (Nottingham, UK) according to the Affymetrix protocols. This array is a whole-genome array, containing 1.2 million probesets of four Perfect Match (PM) probes each. A total of 36 arrays were hybridised (3 mice X 2 genotypes (WT and PTEN*null*) X 3 diets X 2 time-points). Data were analysed using R/Bioconductor [60] and the *aroma.affymetrix *package [18]. Linear probe level models were fit to RMA-background corrected and quantile normalised data to get gene- or exon-level summaries. For annotation we used the current custom CDF file available at the *aroma.affymetrix *website containing the core probesets (17,831 transcript clusters; 224,053 probesets). One array was identified as an outlier and was removed from further analysis. Subsequent statistical data analysis to identify differentially expressed genes was performed using *limma *[61], and evaluation of alternative splicing using *FIRMA *[17] packages. Genes were identified as differentially expressed at different Benjamini and Hochberg adjusted p-values. Alternative splicing events were identified if the difference between FIRMA scores of two groups was statistically significant after Benjamini and Hochberg adjustment. For the plotting of probeset-level data for FIRMA analyses of exon array data we used the Bioconductor *GenomeGraphs *package [62]. To identify pathways that were the most over-presented in the lists of differentially expressed or alternative spliced genes, functional analyses using MAPPFinder and GenMAPP v2.1 were performed http://www.genmapp.org/[63]. The Database for Annotation, Visualization and Integrated Discovery v6 (DAVID; http://david.abcc.ncifcrf.gov/) [64] was used to identify Gene Ontology (GO) categories associated with specific gene lists (Additional file 2 - Table S2; Additional file 3 - Table S3). Microarray data generated in this study are compliant to MIAME criteria and are publicly available through ArrayExpress (Accession E-MEXP-2469).

### PCR of the DMBT1 gene

Total RNA from all the mice was reverse transcribed into cDNA using random primer synthesis from the High-Capacity cDNA Reverse Transcription kit according to the manufacturer's instructions (Applied Biosystems, UK). Primer pairs for the locus flanking exon 2 were 5'- TTGTGGGGTCAAATTCTGTCT-3' and 5'-CTCCAGCATCTTCCTGGTGT-3' and for the locus flanking exon 7 were 5'-CTCAAACAAGCAGTCCCACA-3' and 5'-GTCCCTCCTGGATTCCACC-3'. PCR reactions were carried out in a total volume of 50 μl consisting of 1× green GoTaq^® ^Flexi PCR Buffer, 0.2 mM dNTPs, 0.2 μM primer, 1 mM MgCl_2_, 0.025 units of GoTaq^® ^Flexi DNA Polymerase (Promega, UK). Cycling conditions consisted of 2 min at 95°C for initial denaturation, 30 cycles of denaturation for 30 sec at 95°C, annealing for 1 min at 60°C for exon 2 and 59°C for exon 7, and extension for 1 min at 72°C, followed by 5 min final extension at 72°C. Amplified products were separated in 2% (w/v) agarose gels using 1 μg of the 50 bp DNA ladder (Invitrogen, UK) as a size marker and visualised under UV following ethidium bromide staining.

### Statistical analysis of human orthologues

To estimate whether the overlap between human orthologues of mouse genes induced by SF and human genes induced by a dietary intervention was significant, we initially assumed that the total number of probes from the human Affymetrix U133 Plus 2.0 array and equivalent probes from the mouse Affymetrix Exon 1.0 ST array would be similar. We then performed a Monte Carlo simulation to assess the number of expected possible probes that both change significantly in the mouse (measured probability = 0.000467) and the human (measured probability = 0.10716). This expected number was compared to the measured number using a Binomial test in R [65].

## Abbreviations

FIRMA: Finding Isoforms using Robust Multichip Analysis; HGPIN: high-grade prostatic intraepithelial neoplasia; HSF: high sulforaphane; LSF: low sulforaphane; mPIN: murine prostatic intraepithelial neoplasia; SF: sulforaphane.

## Competing interests

The authors declare that they have no competing interests.

## Authors' contributions

MHT designed and performed the animal study, analysed the array data, performed the splicing experiment. CAS performed the mouse immunohistochemistry. JFD performed the human immunohistochemistry. AM performed the cell analyses. RYB assessed the mouse and human prostate sections. RDM collected the human prostate tissue. RFM conceived the study and together with MHT prepared the manuscript. All authors read and approved the final manuscript.

## Supplementary Material

Additional file 1 **Supplementary Table S1**. Expression of the five-week 'PTEN signature gene' list comprising of 198 genes in all three diets. Click here for file

Additional file 2 **Supplementary Table S2**. Gene ontology categories associated with the 221 genes that were uniquely changed in five week old mice by the LSF diet in the PTEN null compared to WT mice on control diet. Click here for file

Additional file 3 **Supplementary Table S3**. Gene ontology categories associated with the 164 genes in eight-week-old mice as a result of the PTEN deletion. Click here for file

Additional file 4 **Supplementary Table S4**. Functional analysis using GenMAPP of the 1997 transcripts induced by SF in addition to the PTEN signature genes in eight week old mice. Click here for file

Additional file 5 **Supplementary Table S5**. Expression of cell cycle-related genes from the GenMAPP annotation that change in eight week old PTEN null mice on high SF diet. Click here for file

Additional file 6 **Supplementary Table S6**. Expression of apoptosis-related genes from the GenMAPP annotation that change in eight week old PTEN null mice on high SF diet. Click here for file

Additional file 7 **Supplementary Table S7**. Significant FIRMA scores in eight week old PTEN null mice fed SF diets compared to their WT littermates on control diet. Click here for file

Additional file 8 **Supplementary Table S8**. Functional analysis of exons alternatively spliced between eight week old PTEN null mice on low or high SF diets and WT mice on control diet. Click here for file
