# Supplementary material for: The dietary isothiocyanate sulforaphane modulates gene expression and alternative gene splicing in a PTEN null preclinical murine model of prostate cancer
Source: Mol Cancer. 2010 Jul 13;9:189. doi: 10.1186/1476-4598-9-189 (PMC3098008; doi:10.1186/1476-4598-9-189)
Supplement: Additional file 1 — Supplementary Table S1. Expression of the five-week 'PTEN signature gene' list comprising of 198 genes in all three diets. [file 1476-4598-9-189-S1.RTF]

Supplementary Table S1. Expression of the five-week 'PTEN signature gene' list comprising of 198 genes in all three diets 
Transcipt ID	Gene Name	Gene Symbol	Control*	lowSF*	highSF*	
6801914	glutathione peroxidase 2	Gpx2  	3.97	2.55	2.41	
6995177	transmembrane protease, serine 4 	Tmprss4  	2.18	1.57	1.20	
6792838	secreted and transmembrane 1B	Sectm1b  	2.59	1.17	2.14	
6755241	immunoglobulin superfamily, member 9 	Igsf9  	0.86	0.52	0.32	
6832714	submandibular gland protein C	Smgc  	1.78	1.94	1.25	
6836839	solute carrier family 39 (zinc transporter), member 4	Slc39a4  	1.81	1.26	1.49	
6900388	proline/serine-rich coiled-coil 1	Psrc1  	0.94	0.61	0.22	
6967061	serum amyloid A 1 	Saa1  	3.03	1.53	1.92	
6960483	serum amyloid A 2	Saa2  	1.76	1.19	1.02	
6782274	purinergic receptor P2X, ligand-gated ion channel, 1	P2rx1  	-1.72	-1.72	-1.67	
6894304	sterile alpha motif domain containing 10	Samd10  	0.84	0.60	0.06	
6789541	spinster homolog 2 (Drosophila)	Spns2  	1.25	0.62	0.67	
6783041	extracellular proteinase inhibitor	Expi  	4.55	3.37	3.48	
6880033	lysophosphatidylcholine acyltransferase 4	Lpcat4  	1.55	1.08	0.76	
6940039	ankyrin repeat domain 56	Ankrd56  	0.84	0.62	0.16	
6774264	DNA-damage-inducible transcript 4	Ddit4  	1.39	1.26	1.10	
7012305	dystrophin, muscular dystrophy	Dmd  	-1.12	-0.75	-0.83	
6904856	protocadherin 18	Pcdh18  	-0.94	-0.51	-0.33	
6752148	serine (or cysteine) peptidase inhibitor, clade B (ovalbumin), member 11	Serpinb11  	2.49	3.04	1.97	
6925562	zinc finger and BTB domain containing 8b	Zbtb8b  	0.75	0.49	0.37	
6967002	secretory blood group 1 	Sec1  	2.08	1.99	1.32	
6854260	tumor necrosis factor receptor superfamily, member 12a	Tnfrsf12a  	1.35	0.95	0.69	
6855087	tumor necrosis factor	Tnf  	1.95	1.31	0.89	
6850258	tripartite motif-containing 31	Trim31  	1.61	0.76	0.80	
6987439	calponin 1	Cnn1  	-1.17	-1.09	-1.32	
6791338	keratin 23	Krt23  	1.79	1.64	1.08	
6750625	desmin	Des  	-1.32	-0.88	-1.09	
6966934	reticulocalbin 3, EF-hand calcium binding domain	Rcn3  	-1.04	-1.03	-0.69	
6888296	calcitonin receptor-like	Calcrl  	-1.53	-1.04	-0.80	
6872616	protein kinase, cGMP-dependent, type I	Prkg1  	-1.05	-0.87	-0.49	
6870068	transmembrane protein 180	Tmem180  	0.66	0.31	0.29	
6840094	fetuin beta	Fetub  	0.77	0.55	0.28	
6986737	matrix metallopeptidase 7	Mmp7  	2.77	3.04	1.78	
6975871	PDZ and LIM domain 3	Pdlim3  	-1.45	-1.06	-1.35	
6968041	desmuslin	Dmn  	-1.08	-0.76	-0.98	
6992164	WD repeat domain 51A	Wdr51a  	0.69	0.31	0.27	
6762321	ATPase, Ca++ transporting, plasma membrane 4	Atp2b4  	-1.14	-1.05	-0.87	
6789166	ubiquitin specific peptidase 43	Usp43  	0.79	0.63	0.21	
6783818	tubulin tyrosine ligase-like family, member 6	Ttll6  	0.72	0.03	0.09	
6838693	keratin 8	Krt8  	1.14	1.11	0.56	
6963590	spondin 1, (f-spondin) extracellular matrix protein	Spon1  	-0.82	-1.51	-0.99	
6950394	G protein-coupled receptor, family C, group 5, member A	Gprc5a  	2.06	1.18	1.00	
6911013	proline/serine-rich coiled-coil 1	Psrc1  	0.74	0.51	0.15	
6955035	actin, gamma 2, smooth muscle, enteric	Actg2  	-1.49	-1.26	-1.39	
6858563	supervillin	Svil  	-0.73	-0.69	-0.74	
6814956	versican	Vcan  	-1.06	-0.67	-0.80	
6753110	NUAK family, SNF1-like kinase, 2	Nuak2  	0.66	0.47	0.28	
6849312	NADPH oxidase organizer 1	Noxo1  	1.73	1.18	0.95	
6869635	ectonucleoside triphosphate diphosphohydrolase 1 	Entpd1  	-0.85	-0.74	-0.58	
6962577	RAB30, member RAS oncogene family	Rab30  	-0.84	-0.75	-0.19	
6787602	fatty acid binding protein 6, ileal (gastrotropin)	Fabp6  	1.06	0.52	0.32	
6968772	calcium and integrin binding 1 (calmyrin)	Cib1  	0.94	0.73	0.13	
6932379	chemokine (C-X-C motif) ligand 2	Cxcl2  	1.93	1.30	0.38	
6964036	protein kinase C, beta /acid phosphatase 1, soluble 	Prkcb/Acp1	-0.70	-0.74	-0.73	
6881455	phospholipase C, beta 4	Plcb4  	-1.00	-0.78	-0.61	
6941657	2'-5' oligoadenylate synthetase 1A	Oas1a  	1.55	1.14	1.09	
6933998	2'-5' oligoadenylate synthetase 1F	Oas1f  	0.81	0.64	0.11	
6765973	F-box protein 30 	Fbxo30  	-1.00	-0.50	-0.13	
6929865	hepatocyte growth factor activator	Hgfac  	0.78	0.31	0.07	
6806958	asporin	Aspn  	-1.37	-1.64	-0.23	
6972491	cyclin D1	Ccnd1  	-0.76	-0.71	-0.70	
7020286	melanoma antigen, family H, 1	Mageh1  	-0.98	-0.34	-0.50	
6963040	interleukin 18 binding protein	Il18bp  	0.61	0.38	0.30	
6831254	KH domain containing, RNA binding, signal transduction associated 3	Khdrbs3  	-0.56	-0.09	0.00	
6921161	tropomyosin 2, beta	Tpm2  	-1.04	-0.42	-0.90	
6958063	ATP-binding cassette, sub-family C (CFTR/MRP), member 9	Abcc9  	-1.21	-0.86	-0.62	
6755240	SLAM family member 9	Slamf9  	0.77	0.29	0.23	
6963029	interleukin 18 binding protein	Il18bp  	0.60	0.31	0.30	
6924583	solute carrier family 5 (sodium/glucose cotransporter), member 9	Slc5a9  	1.43	0.88	0.74	
6972192	interferon regulatory factor 7	Irf7  	1.66	0.72	0.97	
6840694	myosin, light polypeptide kinase	Mylk  	-1.36	-1.14	-1.22	
6956902	calcium channel, voltage-dependent, L type, alpha 1C subunit	Cacna1c  	-0.83	-0.78	-0.74	
6792839	secreted and transmembrane 1A	Sectm1a  	1.21	0.69	0.46	
6917496	sestrin 2	Sesn2  	0.73	0.17	-0.03	
7017585	pregnancy upregulated non-ubiquitously expressed CaM kinase	Pnck  	-0.89	-0.82	-0.99	
6991192	5' nucleotidase, ecto	Nt5e  	-1.17	-1.36	-0.89	
6857639	solute carrier family 8 (sodium/calcium exchanger), member 1	Slc8a1  	-1.15	-0.82	-0.66	
6872785	NM_001110517		3.42	2.27	2.84	
6900082	tetraspanin 2	Tspan2 	-0.95	-1.12	-0.53	
6820472	epithelial stromal interaction 1 (breast)	Epsti1  	1.30	0.85	0.56	
7015411	coiled-coil domain containing 120	Ccdc120  	0.82	0.49	0.04	
Transcipt ID	Gene Name	Gene Symbol	Control*	lowSF*	highSF*	
6877932	kelch-like 23 (Drosophila)	Klhl23  	-1.01	-0.71	-0.46	
6757898	lung-inducible neuralized-related C3HC4 RING domain protein	Lincr  	1.09	0.37	0.49	
6747696	X Kell blood group precursor related family member 9 homolog	Xkr9  	0.66	0.32	0.41	
6823041	calcium/calmodulin-dependent protein kinase II gamma	Camk2g  	-0.57	-0.45	-0.40	
6806791	CAP, adenylate cyclase-associated protein, 2 (yeast)	Cap2  	-1.65	-0.86	-0.84	
6849595	cyclin-dependent kinase inhibitor 1A (P21)	Cdkn1a  	1.04	0.70	0.12	
6753400	ladinin	Lad1  	0.76	0.67	0.03	
6760292	delta/notch-like EGF-related receptor	Dner  	-1.19	-1.25	-0.71	
6928909	calcium channel, voltage-dependent, alpha2/delta subunit 1 	Cacna2d1  	-0.87	-0.67	-0.54	
6909520	ENSMUST00000070741		1.06	0.96	0.53	
6792097	solute carrier family 16 (monocarboxylic acid transporters), member 6	Slc16a6  	0.75	0.05	-0.05	
6819596	sacsin	Sacs  	-1.29	-1.22	-0.54	
6965947	chemokine (C-X-C motif) ligand 17	Cxcl17  	1.76	1.21	0.46	
6936076	a disintegrin and metallopeptidase domain 22	Adam22  	-0.64	-0.92	-0.50	
6787819	T-cell immunoglobulin and mucin domain containing 2	Timd2  	0.60	0.34	0.55	
6967060	serum amyloid A 4	Saa4  	0.87	0.47	0.36	
6977027	Jun proto-oncogene related gene d	 Jund  	0.79	0.26	0.01	
6781580	transient receptor potential cation channel, subfamily V, member 2	Trpv2	-0.85	-0.83	-0.52	
6966354	hepsin	Hpn  	0.93	0.63	0.23	
6949535	interleukin 17 receptor A	Il17ra  	0.53	0.09	-0.01	
6780745	olfactory receptor 1383	Olfr1383  	1.26	0.84	0.95	
7013967	basic helix-loop-helix domain containing, class B9	Bhlhb9  	-0.91	-0.37	0.31	
6984473	plasma membrane proteolipid	Pllp  	0.60	0.26	-0.14	
6782655	dehydrogenase/reductase (SDR family) member 13	Dhrs13  	0.89	0.32	-0.03	
6892055	ENSMUST00000109896		0.53	0.56	0.16	
6772815	arginase, liver	Arg1  	2.44	1.84	2.62	
6869436	HECT domain containing 2	Hectd2  	-0.58	-0.79	-0.43	
6952298	leucine rich repeat containing 4	Lrrc4  	0.58	0.09	-0.17	
6869222	phosphatase and tensin homolog	Pten  	-0.55	-0.61	-0.30	
6765734	zinc finger CCCH type containing 12D	Zc3h12d  	0.92	0.66	0.28	
6931168	ENSMUST00000081747		1.32	0.96	0.38	
6885616	 ENSMUST00000113912		0.90	0.25	0.31	
6777190	leucine rich repeat containing G protein coupled receptor 5	Lgr5  	-0.93	-0.86	-0.94	
6996667	lipase, hepatic	Lipc  	0.52	0.32	0.15	
6870063	nuclear factor of kappa light polypeptide gene enhancer in B-cells 2, p49/p100	Nfkb2  	0.64	0.27	0.18	
6953763	FK506 binding protein 14	Fkbp14  	-0.86	-0.45	0.03	
6775185	collagen, type VI, alpha 1	Col6a1  	-0.78	-1.24	-0.53	
6845992	ENSMUST00000036732		1.48	1.35	0.62	
6844112	myosin, heavy polypeptide 11, smooth muscle	Myh11  	-1.18	-1.00	-1.11	
6785609	smoothelin	Smtn  	-0.83	-0.76	-0.93	
6936760	protein kinase, AMP-activated, gamma 2 non-catalytic subunit	Prkag2  	-0.63	-0.49	-0.52	
6906634	doublecortin-like kinase 2	Dclk2  	-0.51	-0.63	-0.33	
6870956	T-cell, immune regulator 1, ATPase, H+ transporting, lysosomal V0 protein A3	Tcirg1  	0.59	0.20	0.24	
6836325	transmembrane protein 71	Tmem71  	0.94	0.36	0.81	
6892579	transglutaminase 2, C polypeptide	Tgm2  	-0.67	-0.65	-0.43	
6967001	fucosyltransferase 2	Fut2  	2.52	2.23	1.82	
6994625	cDNA sequence BC004728		0.67	0.18	0.22	
7007728	RIKEN cDNA B230220N19 gene		1.07	0.92	0.68	
6959695	androgen binding protein alpha	Abpa  	1.89	1.36	1.24	
6832256	BCL2-interacting killer	Bik  	0.62	0.45	0.03	
6782859	transmembrane protein 98	Tmem98  	0.73	0.44	0.03	
6751103	chemokine (C-C motif) ligand 20	Ccl20  	1.42	1.13	0.72	
6831775	apolipoprotein L 9b	Apol9b  	1.22	0.64	0.31	
6872290	phosphoglucomutase 5	Pgm5  	-0.76	-0.79	-1.13	
6939931	betacellulin, epidermal growth factor family member	Btc  	0.70	0.51	0.43	
6996448	tropomyosin 1, alpha	Tpm1  	-0.65	-0.31	-0.75	
6764435	thymoma viral proto-oncogene 3	Akt3  	-0.97	-0.78	-0.39	
6921379	tripartite motif-containing 14	Trim14  	0.55	0.09	0.09	
6794572	ets variant gene 1	Etv1  	-0.66	-0.63	-0.36	
6912644	interferon kappa	Ifnk  	0.79	0.40	0.02	
6980941	NIMA (never in mitosis gene a)-related expressed kinase 5	Nek5  	0.57	0.27	0.03	
6803120	coiled-coil domain containing 88C	Ccdc88c  	0.69	0.56	0.23	
6966041	Sh3kbp1 binding protein 1	Shkbp1  	0.59	0.38	0.15	
6852229	latent transforming growth factor beta binding protein 1	Ltbp1  	-0.77	-1.02	-0.68	
6931001	protocadherin 7	Pcdh7  	-1.02	-0.98	-0.36	
6941647	2'-5' oligoadenylate synthetase 2 	Oas2  	1.45	0.37	0.71	
6748535	protein tyrosine phosphatase, non-receptor type 18	Ptpn18  	0.92	0.73	0.35	
6974832	G protein-coupled receptor 124	Gpr124  	-0.61	-0.76	-0.78	
6987308	olfactory receptor 869	Olfr869  	1.10	0.48	0.15	
6899254	small conductance calcium-activated channel, subfamily N, member 3	Kcnn3  	-0.63	-0.55	-0.66	
6875702	ENSMUST00000114336		0.76	0.32	-0.12	
6942563	HIV-1 Rev binding protein-like	Hrbl  	0.57	0.17	0.26	
6869786	RIKEN cDNA 0610010D20 gene		0.58	0.31	0.10	
7015375	chloride channel 5	Clcn5  	-0.59	-0.46	-0.25	
6978263	guanine nucleotide binding protein, alpha O	Gnao1  	-1.17	-1.00	-0.59	
6815268	coagulation factor II (thrombin) receptor-like 1	F2rl1  	1.27	1.20	0.48	
6783997	protein phosphatase 1, regulatory (inhibitor) subunit 1B	Ppp1r1b  	1.55	1.03	0.66	
6958984	dystrophia myotonica-protein kinase	Dmpk  	-0.96	-0.95	-0.68	
6854335	amidohydrolase domain containing 2	Amdhd2  	0.71	0.23	0.36	
6846670	Eph receptor A3	Epha3  	-0.91	-0.59	-0.70	
6901671	endomucin	Emcn  	-1.12	-0.93	-0.64	
6775451	tight junction protein 3	Tjp3  	0.93	0.46	0.28	
Transcipt ID	Gene Name	Gene Symbol	Control*	lowSF*	highSF*	
6983639	Hedgehog-interacting protein	Hhip  	-0.92	-0.94	-0.84	
6760009	serine (or cysteine) peptidase inhibitor, clade E, member 2	Serpine2  	-1.00	-1.02	-0.89	
6892376	glutathione synthetase	Gss  	0.69	0.85	0.47	
6837785	mitogen-activated protein kinase 11	Mapk11  	0.55	0.25	0.14	
6892799	junctophilin 2	Jph2  	-0.93	-0.89	-1.01	
6908684	polypyrimidine tract binding protein 2	Ptbp2  	-0.89	-0.71	-0.68	
6838809	integrin alpha 5 (fibronectin receptor alpha)	Itga5  	-0.69	-0.42	-0.60	
6788657	guanylate kinase 1	Guk1  	0.73	0.53	0.13	
6882627	myosin, light polypeptide 9, regulatory	Myl9  	-0.87	-0.97	-1.35	
6941649	2'-5' oligoadenylate synthetase 3	Oas3  	1.19	0.48	0.38	
6990569	bone morphogenetic protein 5	Bmp5  	-2.11	-1.32	-1.17	
6942192	glucuronidase, beta	Gusb  	0.75	0.46	0.36	
6780570	hepatitis A virus cellular receptor 1	Havcr1  	0.79	0.54	0.75	
6992855	integrin alpha 9	Itga9  	-0.86	-1.00	-0.55	
6822154	CD24a antigen	Cd24a  	0.94	0.99	0.54	
6916540	phosphatidylinositol 3 kinase, regulatory subunit, polypeptide 3 (p55)	Pik3r3  	1.31	1.42	1.29	
6824507	ENSMUST00000036972		-1.08	-0.93	-0.64	
6836888	RNA binding motif protein 9	Rbm9  	-0.51	-0.29	-0.31	
6983513	Rho GTPase activating protein 10	Arhgap10  	-0.89	-0.66	-0.64	
6889978	Meis homeobox 2	Meis2  	-0.72	-0.70	-0.72	
6856203	leucine-rich alpha-2-glycoprotein 1	Lrg1  	1.59	0.49	0.65	
6939990	chemokine (C-X-C motif) ligand 10	Cxcl10  	1.09	1.07	0.81	
6907088	ornithine decarboxylase antizyme 3	Oaz3  	0.75	0.19	0.33	
6806039	serine (or cysteine) peptidase inhibitor, clade B, member 1b	Serpinb1b  	0.72	0.66	0.95	
6893004	transformation related protein 53 regulating kinase	Trp53rk  	0.75	0.64	0.19	
6891064	Ras association (RalGDS/AF-6) domain family member 2	Rassf2  	-0.79	-0.81	-0.54	
6847556	a disintegrin-like and metallopeptidase (reprolysin type) with thrombospondin type 1 motif, 1	Adamts1  	-1.04	-0.67	-0.25	
6903454	cytochrome P450, family 7, subfamily b, polypeptide 1	Cyp7b1  	-0.47	0.06	-0.33	
6978369	matrix metallopeptidase 15	Mmp15  	1.10	0.65	0.41	
6850080	neuraminidase 1	Neu1  	0.90	0.53	0.32	
6964039	protein kinase C, beta	Prkcb  	-1.05	-0.47	-0.09	
6843491	UDP-Gal:betaGlcNAc beta 1,3-galactosyltransferase, polypeptide 5	B3galt5  	0.81	0.39	0.11	
6775337	strawberry notch homolog 2 (Drosophila)	Sbno2  	0.55	0.23	0.07	
7012265	transmembrane protein 47	Tmem47  	-0.60	-0.14	-0.13	
6765218	activating transcription factor 3	Atf3  	1.06	0.87	0.84	
*Numbers refer to the difference in the log2 expression of the three diets in five-week old mice of PTEN null background compared to WT on control diet.
