# Supplementary material for: The dietary isothiocyanate sulforaphane modulates gene expression and alternative gene splicing in a PTEN null preclinical murine model of prostate cancer
Source: Mol Cancer. 2010 Jul 13;9:189. doi: 10.1186/1476-4598-9-189 (PMC3098008; doi:10.1186/1476-4598-9-189)
Supplement: Additional file 2 — Supplementary Table S2. Gene ontology categories associated with the 221 genes that were uniquely changed in five week old mice by the LSF diet in the PTEN null compared to WT mice on control diet. [file 1476-4598-9-189-S2.RTF]

Supplementary Table S2. Gene ontology categories associated with the 221 genes that were uniquely changed in five week old mice by the LSF diet in the PTEN null compared to WT mice on control diet. 
Term*	P-value	Adjusted P-value	
GO:0048856~anatomical structure development	3.29E-08	<0.001	
GO:0007275~multicellular organismal development	6.45E-07	0.001	
GO:0048731~system development	1.34E-06	0.002	
GO:0048513~organ development	1.51E-06	0.001	
GO:0006817~phosphate transport	1.77E-06	0.001	
GO:0007155~cell adhesion	5.05E-06	0.003	
GO:0022610~biological adhesion	5.05E-06	0.003	
GO:0065008~regulation of biological quality	1.29E-05	0.008	
GO:0032502~developmental process	1.69E-05	0.009	
GO:0030198~extracellular matrix organization and biogenesis	4.57E-05	0.023	
GO:0005201~extracellular matrix structural constituent	9.93E-10	<0.001	
GO:0030020~extracellular matrix structural constituent conferring tensile strength	1.60E-08	<0.001	
GO:0005509~calcium ion binding	1.73E-05	0.015	
* Only the Biological Processes and Molecular Function components of Gene Ontology (GO) are reported (Adjusted P-value ≤0.05 by Benjamini). 
