# Supplementary material for: The dietary isothiocyanate sulforaphane modulates gene expression and alternative gene splicing in a PTEN null preclinical murine model of prostate cancer
Source: Mol Cancer. 2010 Jul 13;9:189. doi: 10.1186/1476-4598-9-189 (PMC3098008; doi:10.1186/1476-4598-9-189)
Supplement: Additional file 3 — Supplementary Table S3. Gene ontology categories associated with the 164 genes in eight-week-old mice as a result of the PTEN deletion. [file 1476-4598-9-189-S3.RTF]

Supplementary Table S3. Gene ontology categories associated with the 164 genes in eight-week-old mice as a result of the PTEN deletion.
 
Term*	% of genes	P-value	
GO:0051094~positive regulation of developmental process	3.31%	0.0034	
GO:0050793~regulation of developmental process	5.30%	0.0057	
GO:0045597~positive regulation of cell differentiation	2.65%	0.0078	
GO:0045595~regulation of cell differentiation	3.97%	0.0096	
GO:0045649~regulation of macrophage differentiation	1.32%	0.0212	
GO:0051301~cell division	3.97%	0.0316	
GO:0065008~regulation of biological quality	7.28%	0.0335	
GO:0043412~biopolymer modification	13.25%	0.0344	
GO:0006464~protein modification process	12.58%	0.0438	
GO:0050790~regulation of catalytic activity	4.64%	0.0442	
GO:0043687~post-translational protein modification	11.26%	0.0482	
* Only the Biological Processes components of Gene Ontology (GO) are reported (P-value ≤0.05).
