# Supplementary material for: The dietary isothiocyanate sulforaphane modulates gene expression and alternative gene splicing in a PTEN null preclinical murine model of prostate cancer
Source: Mol Cancer. 2010 Jul 13;9:189. doi: 10.1186/1476-4598-9-189 (PMC3098008; doi:10.1186/1476-4598-9-189)
Supplement: Additional file 4 — Supplementary Table S4. Functional analysis using GenMAPP of the 1997 transcripts induced by SF in addition to the PTEN signature genes in eight week old mice. [file 1476-4598-9-189-S4.RTF]

Supplementary Table S4. Functional analysis using GenMAPP of the 1997 transcripts induced by SF in addition to the PTEN signature genes in eight week old mice.
 
MAPP Name	Number Changed*	Number Measured**	Adjusted   P-value***	
Mm_Ribosomal_Proteins	34	63	<0.001	
Mm_Electron_Transport_Chain-COREG	78	298	<0.001	
Mm_Caspase-Cascade-in-Apoptosis_BioCarta-COREG	31	95	0.005	
Mm_Cell-Cycle-Checkpoints_Reactome-COREG	44	157	0.008	
Mm_B_Cell_Receptor_NetPath_12-MEGINT	107	495	0.010	
Mm_Nucleotide_Metabolism-COREG	56	225	0.024	
Mm_ATM-Signaling-Pathway_BioCarta-COREG	25	77	0.028	
Mm_DNA_replication_Reactome	15	37	0.031	
Mm_HIV-I-Nef-negative-effector-of-Fas-and-TNF_BioCarta-COREG	40	148	0.035	
Mm_IL-5_NetPath_17-MEGINT	61	258	0.037	
Mm_B_Cell_Receptor_NetPath_12-COREG	108	528	0.045	
Only pathways with adjusted P-values ≤ 0.05 are shown. 
* Refers to the transcripts from the input transcript list uniquely induced by low and/or high SF diets (Figure 11) that are present in the specific pathway. **Refers to the total number of transcripts that are present in the pathway. ***P-values were calculated in GenMAPP using a non-parametric statistic based on 2000 permutations of the data and further adjusted for multiple testing by Westfall-Young adjustment.
