# Supplementary material for: The dietary isothiocyanate sulforaphane modulates gene expression and alternative gene splicing in a PTEN null preclinical murine model of prostate cancer
Source: Mol Cancer. 2010 Jul 13;9:189. doi: 10.1186/1476-4598-9-189 (PMC3098008; doi:10.1186/1476-4598-9-189)
Supplement: Additional file 5 — Supplementary Table S5. Expression of cell cycle-related genes from the GenMAPP annotation that change in eight week old PTEN null mice on high SF diet. [file 1476-4598-9-189-S5.DOC]

**Supplementary Table S5**. Expression of cell cycle-related genes from the GenMAPP annotation that change in eight week old PTEN null mice on high SF diet.

| **Transcipt ID** | **Gene Name** | **Gene Symbol** | **Fold*** |
| --- | --- | --- | --- |
| 6815558 | cyclin B1 | Ccnb1 | 2.34 |
| 6791298 | topoisomerase (DNA) II alpha | Top2a | 2.16 |
| 6904300 | cyclin A2 | Ccna2 | 2.01 |
| 6964033 | polo-like kinase 1 (Drosophila) | Plk1 | 1.72 |
| 6756394 | NIMA (never in mitosis gene a)-related expressed kinase 2 | Nek2 | 1.61 |
| 6946778 | MAD2 (mitotic arrest deficient, homolog)-like 1 (yeast) | Mad2l1 | 1.56 |
| 6869503 | kinesin family member 11 | Kif11 | 1.51 |
| 6807022 | CDC28 protein kinase regulatory subunit 2 | Cks2 | 1.48 |
| 6994666 | checkpoint kinase 1 homolog (S. pombe) | Chek1 | 1.46 |
| 6774794 | cell division cycle 2 homolog A (S. pombe) | Cdc2a | 1.43 |
| 6762017 | minichromosome maintenance deficient 6 (S. cerevisiae) | Mcm6 | 1.38 |
| 6893486 | aurora kinase A | Aurka | 1.37 |
| 6933459 | CHK2 checkpoint homolog (S. pombe) | Chek2 | 1.36 |
| 6978923 | cadherin 1 | Cdh1 | 1.33 |
| 7012842 | kinesin family member 4 | Kif4 | 1.33 |
| 6936082 | DBF4 homolog (S. cerevisiae) | Dbf4 | 1.32 |
| 6996646 | cyclin B2 | Ccnb2 | 1.32 |
| 6880451 | budding uninhibited by benzimidazoles 1 homolog, beta (S. cerevisiae) | Bub1b | 1.24 |
| 6843343 | chromatin assembly factor 1, subunit B (p60) | Chaf1b | 1.24 |
| 6963049 | ribonucleotide reductase M1 | Rrm1 | 1.19 |
| 6783998 | START domain containing 3 | Stard3 | 1.17 |
| 6810166 | polo-like kinase 2 (Drosophila) | Plk2 | 1.17 |
| 6880497 | RAD51 homolog (S. cerevisiae) | Rad51 | 1.17 |
| 6871476 | flap structure specific endonuclease 1 | Fen1 | 1.11 |
| 6757282 | minichromosome maintenance deficient 3 (S. cerevisiae) | Mcm3 | 1.11 |
| 6924892 | cell division cycle 20 homolog (S. cerevisiae) | Cdc20 | 1.06 |
| 6977261 | minichromosome maintenance deficient 5, cell division cycle 46 (S. cerevisiae) | Mcm5 | 1.06 |
| 6928457 | cyclin-dependent kinase 6 | Cdk6 | 1.01 |
| 6898241 | structural maintenance of chromosomes 4 | Smc4 | 0.98 |
| 6911682 | cyclin E2 | Ccne2 | 0.98 |
| 6942604 | minichromosome maintenance deficient 7 (S. cerevisiae) | Mcm7 | 0.98 |
| 6953331 | enhancer of zeste homolog 2 (Drosophila) | Ezh2 | 0.97 |
| 6891070 | proliferating cell nuclear antigen | Pcna | 0.96 |
| 6785307 | baculoviral IAP repeat-containing 5 | Birc5 | 0.96 |
| 6831628 | CDC28 protein kinase regulatory subunit 2 | Cks2 | 0.94 |
| 6784054 | cell division cycle 6 homolog (S. cerevisiae) | Cdc6 | 0.93 |
| 6936981 | thymidylate synthase | Tyms | 0.91 |
| 6925054 | cytidine 5'-triphosphate synthase | Ctps | 0.90 |
| 6765235 | denticleless homolog (Drosophila) | Dtl | 0.88 |
| 6955381 | minichromosome maintenance deficient 2 mitotin (S. cerevisiae) | Mcm2 | 0.86 |
| 6769601 | thymidylate synthase, pseudogene | Tyms-ps | 0.75 |
| 6924801 | nuclear autoantigenic sperm protein (histone-binding) | Nasp | 0.74 |
| 6966600 | cyclin E1 | Ccne1 | 0.74 |
| 6879915 | kinesin family member 18A | Kif18a | 0.72 |
| 6866631 | MAD homolog 4 (Drosophila) | Smad4 | 0.70 |
| 6757732 | DNA primase, p58 subunit | Prim2 | 0.70 |
| 6933422 | polymerase (DNA directed), epsilon | Pole | 0.70 |
| 6753592 | asp (abnormal spindle)-like, microcephaly associated (Drosophila) | Aspm | 0.69 |
| 6844177 | minichromosome maintenance deficient 4 homolog (S. cerevisiae) | Mcm4 | 0.67 |
| 6801454 | polymerase (DNA directed), epsilon 2 (p59 subunit) | Pole2 | 0.66 |
| 6996440 | RAB8B, member RAS oncogene family | Rab8b | 0.64 |
| 6892505 | retinoblastoma-like 1 (p107) | Rbl1 | 0.61 |
| 6833919 | S-phase kinase-associated protein 2 (p45) | Skp2 | 0.59 |
| 6896804 | exosome component 9 | Exosc9 | 0.59 |
| 6749720 | Ngg1 interacting factor 3-like 1 (S. pombe) | Nif3l1 | 0.59 |
| 6828573 | nucleoporin 155 | Nup155 | 0.57 |
| 6778055 | cyclin-dependent kinase 2 | Cdk2 | 0.55 |
| 6977142 | RAB8A, member RAS oncogene family | Rab8a | 0.54 |
| 6797772 | vaccinia related kinase 1 | Vrk1 | 0.49 |
| 6919685 | integrator complex subunit 8 | Ints8 | 0.41 |
| 6929907 | polo-like kinase 1 (Drosophila) | Plk1 | 0.25 |
| 6919191 | dishevelled, dsh homolog 1 (Drosophila) | Dvl1 | -0.46 |
| 6791543 | histone deacetylase 5 | Hdac5 | -0.49 |
| 6839930 | dishevelled 3, dsh homolog (Drosophila) | Dvl3 | -0.55 |
| 6957263 | cyclin D2 | Ccnd2 | -0.80 |
| 6936750 | SWI/SNF related, matrix associated, actin dependent regulator of chromatin, subfamily d, member 3 | Smarcd3 | -1.32 |

* Log_2_ fold change compared to eight week old WT mice on control diet (adjusted P≤0.05).
