# Supplementary material for: The dietary isothiocyanate sulforaphane modulates gene expression and alternative gene splicing in a PTEN null preclinical murine model of prostate cancer
Source: Mol Cancer. 2010 Jul 13;9:189. doi: 10.1186/1476-4598-9-189 (PMC3098008; doi:10.1186/1476-4598-9-189)
Supplement: Additional file 6 — Supplementary Table S6. Expression of apoptosis-related genes from the GenMAPP annotation that change in eight week old PTEN null mice on high SF diet. [file 1476-4598-9-189-S6.DOC]

**Supplementary Table S6**. Expression of apoptosis-related genes from the GenMAPP annotation that change in eight week old PTEN null mice on high SF diet.

| **Transcipt ID** | **Gene Name** | **Gene Symbol** | **Fold*** |
| --- | --- | --- | --- |
| 6815558 | cyclin B1 | Ccnb1 | 2.33 |
| 6791298 | topoisomerase (DNA) II alpha | Top2a | 2.16 |
| 6904300 | cyclin A2 | Ccna2 | 2.01 |
| 6946778 | MAD2 (mitotic arrest deficient, homolog)-like 1 (yeast) | Mad2l1 | 1.56 |
| 6824880 | granzyme B | Gzmb | 1.36 |
| 6933459 | CHK2 checkpoint homolog (S. pombe) | Chek2 | 1.36 |
| 7012842 | kinesin family member 4 | Kif4 | 1.33 |
| 6996646 | cyclin B2 | Ccnb2 | 1.32 |
| 6913499 | structural maintenance of chromosomes 2 | Smc2 | 1.29 |
| 6963049 | ribonucleotide reductase M1 | Rrm1 | 1.19 |
| 6975913 | caspase 3 | Casp3 | 1.14 |
| 6757282 | minichromosome maintenance deficient 3 (S. cerevisiae) | Mcm3 | 1.11 |
| 6977261 | minichromosome maintenance deficient 5, cell division cycle 46 (S. cerevisiae) | Mcm5 | 1.06 |
| 6870614 | caspase 7 | Casp7 | 1.05 |
| 6942604 | minichromosome maintenance deficient 7 (S. cerevisiae) | Mcm7 | 0.98 |
| 6990295 | a disintegrin and metallopeptidase domain 10 | Adam10 | 0.98 |
| 6891070 | proliferating cell nuclear antigen | Pcna | 0.96 |
| 6955381 | minichromosome maintenance deficient 2 mitotin (S. cerevisiae) | Mcm2 | 0.86 |
| 6757732 | DNA primase, p58 subunit | Prim2 | 0.70 |
| 6933422 | polymerase (DNA directed), epsilon | Pole | 0.70 |
| 6844177 | minichromosome maintenance deficient 4 homolog (S. cerevisiae) | Mcm4 | 0.67 |
| 6801454 | polymerase (DNA directed), epsilon 2 (p59 subunit) | Pole2 | 0.66 |
| 6896804 | exosome component 9 | Exosc9 | 0.59 |
| 6919191 | dishevelled, dsh homolog 1 (Drosophila) | Dvl1 | -0.45 |
| 6839930 | dishevelled 3, dsh homolog (Drosophila) | Dvl3 | -0.55 |
| 6876014 | Rap guanine nucleotide exchange factor (GEF) 1 | Rapgef1 | -0.68 |

* Log_2_ fold change compared to eight week old WT mice on control diet (adjusted P≤0.05).
