# Supplementary material for: The dietary isothiocyanate sulforaphane modulates gene expression and alternative gene splicing in a PTEN null preclinical murine model of prostate cancer
Source: Mol Cancer. 2010 Jul 13;9:189. doi: 10.1186/1476-4598-9-189 (PMC3098008; doi:10.1186/1476-4598-9-189)
Supplement: Additional file 7 — Supplementary Table S7. Significant FIRMA scores in eight week old PTEN null mice fed SF diets compared to their WT littermates on control diet. [file 1476-4598-9-189-S7.RTF]

Supplementary Table S7. Significant FIRMA scores in eight week old PTEN null mice fed SF diets compared to their WT littermates on control diet.
Control diet
ProbeSet ID	Gene Symbol	Fold*	Adjusted 
P-value**	
4823291	Pten	-2.9	0.024	
4939702	LOC544904 /LOC630348	4.1	0.024	
LowSF diet
ProbeSet ID	Gene Symbol	Fold*	Adjusted 
P-value**	ProbeSet ID	Gene Symbol	Fold*	Adjusted 
P-value**	ProbeSet ID	Gene Symbol	Fold*	Adjusted 
P-value**	
5452304	Cic 	-2.94	0.006	4691944	Ran	-2.44	0.023	4756111	Ms4a4b	2.58	0.040	
5509779	Mrps18b	-3.39	0.006	4463623	Slc44a4	2.20	0.023	4727463	Cdh17	-2.19	0.040	
5296122	Cpne9	3.04	0.006	4783139	Svil	-4.72	0.023	4329207	Cxadr	3.17	0.040	
5229594	Fth1	-6.26	0.006	4652291	Ssr4	-3.51	0.023	5225216	Tff3	2.03	0.040	
4732701	Selenbp1	-2.78	0.006	5315879	Noxa1	3.81	0.024	4734602	XM_984165	1.69	0.040	
4352551	Ndufa4	-7.11	0.006	5497118	Krt15	-2.94	0.024	5308566	Gsdmc1	1.78	0.041	
5462183	Rps18	-9.69	0.006	5406748	---	-2.94	0.024	4339917	Mylc2b	-4.18	0.041	
5055646	Krt5	3.21	0.006	4704333	Napsa	-1.70	0.024	4555411	Rrp1	-3.15	0.041	
4601869	Uqcrh 	2.65	0.006	5571154	---	2.01	0.024	5166374	Zcchc18	-2.47	0.041	
5025889	Eef2	-7.02	0.006	4316366	H2-Eb1	-4.51	0.024	5403558	Spp1	-4.59	0.041	
5400457	Serbp1	-4.46	0.006	4980087	Atp5o	1.84	0.025	4915387	Sgpl1	-1.76	0.041	
5553941	Boc	-5.24	0.007	5538705	Cxcl10	-2.83	0.025	4705190	Sord	3.61	0.041	
4867791	Gpsn2	-6.18	0.007	5599369	---	-6.55	0.025	4690522	Prdx5	-4.38	0.041	
5512678	Psmc1	-4.85	0.008	4871315	Ccnb1	2.54	0.027	5472056	Serpina3c	3.70	0.041	
5005201	Dmbt1	-4.14	0.008	5504097	Sbno2	-2.36	0.028	5265086	Top2b	-3.05	0.041	
4309824	Dmbt1	-2.90	0.008	5455907	Oasl2	-4.35	0.028	4981168	Fis1	-5.48	0.041	
4743987	ENSMUST00000021311	2.44	0.008	5208534	Zbp1	-2.15	0.028	4855523	Fuca1	-2.89	0.041	
5254312	Slc6a9	-3.66	0.008	4529638	Dnajc7	-3.12	0.029	5486083	Mcoln2	-3.09	0.041	
4860777	Irf7	-6.14	0.008	5313079	Rpl37	-3.77	0.029	5186747	Car3	1.79	0.041	
5341082	Oas2	-2.92	0.008	4677375	Hspa5	-6.56	0.029	5483267	Ptdss2	2.28	0.041	
5308767	Rps4x	2.90	0.008	4587626	Rpl10a	2.56	0.029	4938043	Tufm	-2.94	0.041	
5530014	Tff3	-3.11	0.008	4865569	Oit1	-4.18	0.029	4735947	Qsox1	-3.58	0.041	
5409490	Cox8a	-2.37	0.009	5277201	D10Jhu81e	-3.64	0.029	4923780	Ctage5	-3.61	0.041	
4363323	Akr1b3	-6.12	0.010	4887361	---	-3.67	0.029	4730779	Nfam1	-1.86	0.041	
5130487	Pcolce	-4.17	0.010	5285517	Tpt1	-2.96	0.030	4920016	Rpl37a	-2.15	0.041	
4421402	Sfrp4	-4.48	0.010	5359385	Rsad2	-2.54	0.030	4669905	Phpt1	-3.28	0.041	
4961546	Gpd1	-7.42	0.010	4309579	Polr2g	-2.83	0.030	5183258	Wdr67	-2.46	0.041	
5426558	Atp5g2	-5.02	0.010	4837199	Aof1	4.52	0.030	5524642	Slmap	-2.56	0.042	
4834127	Rps25	-4.37	0.011	4342146	Rgr	-2.33	0.030	5030604	Actr1a	-4.55	0.042	
4363227	ENSMUSG00000059776	-4.25	0.011	4743508	Naip1	-3.48	0.030	5237383	Slc39a8	-7.22	0.042	
5521471	Dmbt1	-4.04	0.011	4433586	Rps8	-5.79	0.030	5388048	Cope	-5.98	0.042	
5103131	Rps8	-5.76	0.011	4737085	Ahsa1	-3.11	0.030	5570275	Pop5	-2.93	0.043	
5260375	Cfd	-5.63	0.011	5505888	Ubap2l	-4.27	0.030	4463650	Aldh2	-5.02	0.043	
4481466	Expi	1.59	0.011	4433492	Igsf6	-2.09	0.030	4965910	Rplp0	-5.05	0.043	
5198023	Prpf6	-4.32	0.011	5099660	Mdh1	-3.68	0.030	5607244	Psma6	-5.89	0.043	
4729600	Rpl9	-3.49	0.011	5491126	Sparcl1	-4.89	0.030	5015151	Dgcr2	-3.40	0.045	
5034637	ENSMUST00000103472	3.63	0.011	4411985	Amica1	2.09	0.030	4696367	Adrm1	-3.61	0.045	
4837465	Apol9b	2.98	0.011	5226044	Sec14l2	2.03	0.030	4872995	Smarce1	-2.37	0.045	
5400162	Lipe	-7.84	0.011	4676903	Nudc	-5.54	0.030	5592906	Lgals9	-2.00	0.045	
5297064	Rpl9	2.45	0.011	5439150	Nt5c	2.43	0.030	4582109	ENSMUST00000103472	4.85	0.045	
5037049	Ube2c	2.06	0.011	5124065	Melk	1.55	0.030	4595709	Sod1	-7.29	0.045	
5088297	Tspo	-4.12	0.012	4980021	Flii	4.15	0.030	5206369	Rpl35a	-3.78	0.045	
4867103	XR_032753 	5.00	0.012	5352419	Cfb	-4.42	0.030	5208771	Sectm1b	-2.28	0.045	
5118064	S3-12	-4.11	0.012	5267402	ENSMUST00000046207	1.96	0.030	5107231	Slc39a4	-2.09	0.045	
4569481	Rac2	-2.45	0.012	5068982	Eif3h	-3.63	0.030	4985755	Oas2	1.81	0.045	
4561061	Scd1	-8.76	0.012	5390225	XM_986755	3.71	0.030	4328764	Josd2	-3.05	0.045	
4704643	Etfb	-5.96	0.012	4920690	Fut11	3.12	0.030	4760472	Atp1a2	4.97	0.045	
4331476	Rpl10a	-3.07	0.012	5263746	Lyz2	2.32	0.030	4568627	Mthfd1l	-2.19	0.045	
5527126	Calr	-4.64	0.012	4938178	Znrf1	-2.73	0.031	5424032	Tmem8	2.28	0.045	
4402378	Chn2	3.44	0.012	5291795	Kif4	1.52	0.031	4699026	Tns1	-1.89	0.045	
5581730	Muc13	1.99	0.013	5449033	Cyba	-4.86	0.031	5208858	Asf1b	-2.33	0.045	
5345698	Lrg1	2.20	0.013	4939702	ENSMUST00000103469	3.04	0.031	4492100	Lyz1	1.95	0.045	
4500344	Hsd11b2	-3.18	0.013	4504258	Cct3	-4.60	0.031	4319158	---	2.28	0.045	
5606002	Tsnaxip1	-2.64	0.013	4873646	Ube2d3	-2.89	0.031	4499803	Ifi44	-3.23	0.045	
5553020	Cpxm1	-2.96	0.013	5240079	Eif3i	-3.76	0.031	4879461	Ctsb	-3.63	0.045	
4770598	Rpl36al	-7.86	0.013	4624148	Rpl4	-6.96	0.031	5147396	Shisa4	2.02	0.045	
5123052	Slfn1	2.24	0.013	4926448	Cct5	-3.28	0.031	5057258	Gabarap	-1.91	0.045	
4651408	Gabrp	-4.19	0.013	4524485	Lamp2	-3.26	0.031	5327274	Bcl2a1b	-2.29	0.045	
4709162	Abcg1	-3.49	0.013	5222229	Lbp	-6.14	0.031	5107817	Ube2g2	-2.28	0.045	
5270734	 Psma7	-3.55	0.014	5323160	Zmiz2	-2.09	0.032	4863220	Hspb1	-3.41	0.045	
5185263	Pgm5	4.43	0.015	4767737	Slc39a4	-2.89	0.032	5178593	ENSMUST00000114007	-1.83	0.045	
4587061	Pcp4	5.58	0.015	5499759	BC043674	4.66	0.032	4717379	Krt6a	-3.25	0.045	
ProbeSet ID	Gene Symbol	Fold*	Adjusted 
P-value**	ProbeSet ID	Gene Symbol	Fold*	Adjusted 
P-value**	ProbeSet ID	Gene Symbol	Fold*	Adjusted 
P-value**	
4491496	Nt5c 	2.54	0.015	4539655	Gstm1	4.16	0.032	5488305	Clstn1	-5.68	0.045	
5051010	Eef2	-9.41	0.016	5610791	Ngfrap1	-2.47	0.032	4970665	Rps6	2.22	0.045	
4768762	Gns	-5.26	0.016	5480060	Clu	-1.50	0.033	5106138	Lpl	-3.50	0.045	
4305328	Mmp10	2.67	0.016	4622686	Eif3a	-2.93	0.033	4573380	Ube2g1	2.04	0.045	
5300211	Rrbp1	-2.87	0.016	4418040	Naip1	2.52	0.033	4389786	Tbca	-3.32	0.045	
4361708	Fkbp9	-5.44	0.016	4365990	Coq6	-4.28	0.033	4507584	Myl6	2.85	0.045	
4329891	Pnkd	-2.34	0.016	5615699	Calm2	-2.30	0.034	5210948	Vcl	-2.38	0.045	
4650444	Hsd11b2	-3.08	0.017	4487970	Kif20a	-3.60	0.034	4794298	ENSMUST00000111231	-2.41	0.045	
4413412	BC027314 	3.35	0.017	4578660	Prdx4	-3.43	0.034	4931681	Aldoa	-4.16	0.045	
4342131	Dmbt1	-3.47	0.017	4508157	Rpl15	-3.11	0.034	5339673	Atf4	-5.04	0.045	
4404006	Oasl2	-5.04	0.017	5186353	Cenpm	2.96	0.034	5096391	Ptrf	4.80	0.045	
4727405	Tpm2	-3.79	0.019	5538155	Iqgap3	2.27	0.034	4386553	Bub1	2.00	0.045	
4718548	Sval1	-2.44	0.019	5370990	Dhx58	2.24	0.034	4872244	Trim44	-2.50	0.045	
4721489	Hck	2.45	0.019	4947393	Apol9b	-2.36	0.034	5564280	Atp5f1	-4.28	0.045	
4634234	Mmp10	3.85	0.019	5070264	Otub1	-2.96	0.034	5579856	Rpl7	-3.95	0.045	
4445676	Pgm2	-7.09	0.019	5407783	Rpl17	-5.63	0.034	4468762	Nnat	-2.09	0.045	
4575796	Spock2	2.22	0.019	4496471	Arg1	-4.17	0.035	5050559	Tmc5	2.75	0.045	
4533834	Kif20a	1.81	0.019	4519550	Eml3	-2.87	0.035	5500877	Rpn2	-2.60	0.046	
5578447	Rpl37a	-4.80	0.019	5461806	Vat1	-2.98	0.035	5537389	Pls3	-3.52	0.046	
4865409	Krt6b	-3.00	0.019	4678047	Cct5	-3.90	0.036	4867373	Myo1c	-3.63	0.046	
5542244	Tpcn2	1.97	0.020	5423363	Acta2	2.32	0.036	4813337	Oasl2	1.81	0.046	
5533792	Acaa2	-4.09	0.021	5442840	Tsen34	-3.24	0.036	5275765	Cr1l	-4.33	0.046	
4317909	Rplp1	-5.34	0.021	5429817	Erp29	-3.76	0.037	4885129	Clec2e	2.27	0.046	
4807289	Map1lc3a	-3.46	0.021	4754611	Aga	-4.26	0.038	4555295	Krt5	-7.77	0.046	
5122647	Eif3f 	-4.24	0.021	4712893	Cd44	3.71	0.038	4418573	Atp2a2	-6.24	0.047	
4890905	Rrbp1	-2.84	0.021	5049377	Zcrb1	-3.39	0.038	5122514	Ptprc	-2.96	0.048	
5085065	Vkorc1	-6.34	0.023	5232551	Rac2	-3.56	0.038	4880343	Tmprss4	-1.91	0.048	
5255391	ENSMUST00000055447	-3.32	0.023	4770696	Tmod1	-2.15	0.038	4465731	Dmbt1	3.24	0.048	
4578442	Tagln	2.94	0.023	4649161	Mrpl48	-4.05	0.038	4499438	Zfand2a	-2.79	0.048	
4327090	Dmd	1.88	0.023	5069254	Hist1h2be	2.25	0.038	4973148	Adrm1	-3.63	0.048	
4336274	Mbc2	-3.52	0.023	4522250	Lass2	-4.78	0.038	5090486	Reg3g	-3.54	0.050	
4481107	Atp5g2	-5.36	0.023	4847913	Rpl8	-8.66	0.039	5383989	Dtx3l	-4.44	0.050	
5366631	Pbsn	-5.39	0.023	4573629	Rpl35	-3.02	0.039	5423932	ENSMUST00000103469	3.22	0.050	
4871161	Expi	-2.58	0.023	5598794	ENSMUST00000046207	-2.45	0.040					
HighSF diet
ProbeSet ID	Gene Symbol	Fold*	Adjusted
P-value**	ProbeSet ID	Gene Symbol	Fold*	Adjusted 
P-value**	ProbeSet ID	Gene Symbol	Fold*	Adjusted 
P-value**	
5055646	Krt5	4.35	<0.001	5456040	Cldn19	-3.44	0.024	4694892	Sox4	-2.51	0.039	
4893234	Gzmd	3.75	0.001	5226044	Sec14l2	2.12	0.024	5613646	Tmc3	1.79	0.039	
4328335	Gzmd	-4.42	0.001	5413818	Ly6a	5.05	0.024	4435703	Grm6	-1.68	0.039	
4939702	ENSMUST00000103469	4.63	0.001	4471045	Ceacam2	-5.88	0.026	5208534	Zbp1	-2.03	0.039	
4686927	Serpinb1c	-5.04	0.005	4823291	Pten	-2.25	0.026	5466851	Tmprss4	2.80	0.039	
5431630	Gzmf	3.34	0.005	4593722	Oasl1	2.08	0.026	4380919	Col12a1	3.18	0.039	
4735947	Qsox1	-4.98	0.005	4756111	Ms4a4b	2.80	0.026	5381853	Arpc3	3.33	0.039	
4634234	Mmp10	4.69	0.005	5195222	Pgm5	6.32	0.026	4885129	Clec2e	2.37	0.039	
5403558	Spp1	-6.32	0.005	5315879	Noxa1	3.79	0.026	4615486	Ptprc	-2.56	0.039	
4421402	Sfrp4	-4.89	0.005	4340601	Slc15a3	2.39	0.026	4437563	Prkd3	-2.74	0.039	
4651408	Gabrp	-4.88	0.005	4556801	Tnf	-2.39	0.026	4838961	Il10ra	-2.39	0.040	
4305328	Mmp10	3.17	0.005	4350167	Gzmn	4.16	0.026	4917105	Enc1	-2.34	0.040	
4485965	Mfge8	3.92	0.005	5521471	Dmbt1	-3.55	0.026	5537422	ENSMUST00000021164	2.85	0.040	
5341082	Oas2	-3.05	0.005	4386553	Bub1	2.23	0.026	4546068	ENSMUST00000108827	-2.57	0.040	
5439150	Nt5c	3.14	0.005	4817937	ENSMUST00000045802	2.28	0.026	4388786	Bbs7	1.85	0.040	
4539655	Gstm1	5.44	0.005	5222229	Lbp	-6.39	0.026	4358375	Reg3b	-2.99	0.040	
4789109	Ly6a	-2.89	0.005	5496512	Mylk	3.05	0.026	5093863	Tesk1	2.21	0.040	
5530014	Tff3	-3.21	0.005	4644294	Pmaip1	-2.09	0.028	5069254	Hist1h2be	2.24	0.040	
5588014	ENSMUST00000089448	-2.68	0.005	5352062	Atp6v0a4	3.33	0.028	4699472	Mrgprb3	-2.52	0.040	
4496471	Arg1	-5.36	0.006	4860777	Irf7	-5.04	0.028	4394071	Olfml2a	-1.92	0.040	
4481466	Expi	1.70	0.006	4691385	Ceacam2	-5.43	0.028	4881542	Parp9	-4.62	0.040	
5423932	ENSMUST00000103469	4.37	0.007	5061771	Clec2e	-3.04	0.029	4572868	Ccl20	-2.13	0.040	
5494636	Pcdh21	-2.86	0.007	4567619	Krt6b	3.51	0.029	4716129	Rps13	-3.98	0.040	
4837465	Apol9b	3.15	0.007	4517676	Es1	-3.58	0.029	4477147	Birc5	1.62	0.040	
4709162	Abcg1	-3.79	0.009	4581596	Col12a1	3.55	0.029	5146555	Il18bp	1.50	0.041	
4971951	Svil	-3.99	0.009	5307645	Tnc	-3.19	0.030	4499803	Ifi44	-3.30	0.041	
5472056	Serpina3c	4.70	0.009	5590066	Noxa1	-2.87	0.030	5397487	Cdh17	2.77	0.042	
4756091	ENSMUST00000108827	-2.91	0.009	4867744	B4galt5	-2.76	0.030	4900170	Il13ra1	1.90	0.042	
5398073	Cxcl11	-3.74	0.009	4324202	Stfa1/ Stfa3	2.23	0.030	4361997	Il4ra	-2.74	0.042	
5011183	Clec4a3	-3.74	0.010	5034892	Atoh7	2.57	0.030	5135254	Dennd2d	-3.42	0.043	
4505826	Cd24a	-2.12	0.010	4620396	Wdfy1	2.23	0.030	5539598	Cyp4f18	-2.05	0.043	
5483540	Cdh17	-3.02	0.010	5498876	Dvl2	2.09	0.030	4726910	Sgol1	1.98	0.043	
4413412	BC027314	3.63	0.010	5382378	Pogz	1.78	0.030	4729600	Rpl9	-2.83	0.043	
4956143	Frap1	2.72	0.010	5455907	Oasl2	-4.30	0.030	5298803	Tmprss4	-2.47	0.043	
4732701	Selenbp1	-2.44	0.010	4721489	Hck	2.29	0.030	4587061	Pcp4	4.74	0.044	
4500344	Hsd11b2	-3.29	0.011	5077048	ENSMUST00000036972	-2.65	0.030	4808776	Lgals4	-2.24	0.044	
4358591	Dmd	-1.97	0.011	4360696	NM_182807	2.31	0.030	5237661	Rps6ka1	-3.30	0.045	
4965235	Slfn1	-2.62	0.011	5124065	Melk	1.56	0.030	5538705	Cxcl10	-2.58	0.045	
5237383	Slc39a8	-8.84	0.012	5320681	Casc5	-1.72	0.030	5156631	Arhgap20	1.93	0.045	
4365904	Sorbs1	4.32	0.012	4525140	Dhx58	-2.86	0.030	5484825	Ifi203	-2.24	0.045	
ProbeSet ID	Gene Symbol	Fold*	Adjusted
P-value**	ProbeSet ID	Gene Symbol	Fold*	Adjusted 
P-value**	ProbeSet ID	Gene Symbol	Fold*	Adjusted 
P-value**	
4408136	Mrvi1	2.56	0.014	4866638	Mapk8ip1	-2.31	0.030	4491496	Nt5c	2.15	0.045	
5588136	Dmd	2.00	0.014	5090342	Atp4a	2.00	0.030	5123052	Slfn1	1.87	0.045	
5122514	Ptprc	-3.68	0.015	5558517	Pcp4	-2.19	0.030	5593584	Oas1g	-3.14	0.045	
4629304	Vsig1	-2.86	0.016	5392255	ENSMUST00000103469	3.83	0.030	5369413	Sec1	1.55	0.045	
4663945	Lincr	-3.90	0.016	4406822	Tlr2	-2.42	0.030	4636040	Vsig1	-2.07	0.045	
4767737	Slc39a4	-3.29	0.016	5444559	Efhd2	-2.73	0.031	4960789	Gcc1	1.59	0.045	
4880343	Tmprss4	-2.35	0.016	4743987	ENSMUST00000021311	1.96	0.031	4432992	Moxd1	-2.60	0.045	
5037049	Ube2c	1.99	0.016	4967260	Serpinb1c	-4.46	0.031	4947953	Cyfip1	3.48	0.045	
4872707	Phf11	-4.07	0.017	4913530	Bspry	-3.41	0.031	5354158	Chst7	2.16	0.045	
5345698	Lrg1	2.17	0.017	4801182	Muc13	2.24	0.031	5262219	Gsn	2.16	0.045	
5327274	Bcl2a1b	-2.74	0.017	5261163	Arl4c	-3.10	0.031	4568627	Mthfd1l	-2.20	0.045	
5478909	Mmp3	-3.74	0.018	5151075	Spock2	-1.77	0.031	5508390	Gzmf	-2.64	0.045	
5589346	Ccl7	-2.77	0.018	4523201	Alox5ap	-3.85	0.031	4518307	Negr1	3.20	0.045	
4871161	Expi	-2.73	0.018	4707692	Bst2	-4.89	0.031	5297376	Tnp2	-2.21	0.045	
5017846	Msn	-4.26	0.019	5257473	---	2.35	0.031	5308566	Gsdmc1	1.74	0.045	
4985100	Gzmd	2.56	0.019	4631430	Steap1	-2.24	0.032	4465731	Dmbt1	3.30	0.045	
4336783	Best2	-2.22	0.020	5050559	Tmc5	2.96	0.032	5249312	Pole2	2.76	0.045	
4706761	Sval1	-2.40	0.020	4991533	Mylk	2.41	0.032	4873807	Fcgr1	2.46	0.045	
5027505	Sec14l2	-3.94	0.020	4674944	Bace2	-2.45	0.032	5386031	Lbp	-3.81	0.045	
4650444	Hsd11b2	-3.07	0.020	4918720	BC007165	1.88	0.032	4472802	Gzmc	-2.93	0.045	
5321437	Cxcl5	-2.69	0.020	4497754	Vill	-4.14	0.032	4864133	Bmp1	-2.84	0.045	
5502136	ENSMUST00000103469	3.70	0.020	5421678	Lrmp	3.52	0.032	4743508	Naip1	-3.22	0.045	
5090486	Reg3g	-4.21	0.020	4863202	Oas2	-2.86	0.034	5457116	Map2k1	-2.87	0.045	
5301586	Cpxm1	-3.13	0.020	4586895	Adam12	-2.18	0.034	4847433	Pcdh21	-2.38	0.045	
4924273	Atp6v0a4	2.78	0.021	5548312	Ccl4	2.54	0.035	5395088	Hkdc1	1.95	0.045	
5137485	Oas2	-3.32	0.021	4851453	ENSMUST00000089628	-2.21	0.035	4506238	Rcc2	1.91	0.045	
4733439	Fcgr1	-2.59	0.022	5151096	Cd7	2.37	0.035	4670846	Ccna2	1.89	0.045	
4309824	Dmbt1	-2.48	0.022	4966069	Sval1	-3.40	0.035	4947393	Apol9b	-2.25	0.046	
4519550	Eml3	-3.16	0.022	5239940	Npl	-3.18	0.035	4439769	Cytip	-3.09	0.046	
4329207	Cxadr	3.55	0.022	5423363	Acta2	2.34	0.036	5460141	Lrrfip1	2.81	0.046	
4995701	Ablim3	-2.59	0.022	5047733	Atp4a	2.08	0.036	5546770	Il2rb	-2.06	0.047	
5082494	Pgk1	2.63	0.022	5495306	Gdpd2	-2.22	0.036	5353494	Tnip3	-3.40	0.047	
5496663	Blvra	1.79	0.022	4924271	Ms4a4c	-3.01	0.036	5514074	Vwde	-1.89	0.047	
5263746	Lyz2	2.48	0.023	4990206	Ctsw	2.23	0.036	5225216	Tff3	1.97	0.047	
4869518	Ctss	-6.16	0.023	5421408	Indo	-2.07	0.036	5319957	ENSMUST00000089628	-1.90	0.047	
5308767	Rps4x	2.49	0.023	5616961	Pla2g4c	-2.00	0.036	5213656	Gzme	2.42	0.047	
4985755	Oas2	2.05	0.024	5153404	Flt3	-2.05	0.036	4843394	Pole2	-2.19	0.047	
4894200	Frzb	-2.98	0.024	5180056	Nnat	4.13	0.036	4411985	Amica1	1.93	0.047	
4865409	Krt6b	-2.96	0.024	4568859	Ms4a4c	-2.74	0.036	5385538	Akna	-2.18	0.047	
4857433	Saa1	-3.16	0.024	4846784	Myh11	2.64	0.036	5121880	Ptx3	-2.09	0.047	
4920690	Fut11	3.31	0.024	4871315	Ccnb1	2.40	0.037	5291795	Kif4	1.41	0.047	
5453656	Wars	2.05	0.024	5578370	Ppp1r1b	-3.14	0.037	4363942	Car2	-4.89	0.048	
4418040	Naip1	2.71	0.024	5601626	Lair1	2.32	0.038	4861031	Ctnnal1	-1.83	0.048	
5005201	Dmbt1	-3.47	0.024	4325549	Tnfrsf11b	-2.28	0.038	5410824	Polr2d	2.36	0.048	
4641650	Aldh3a1	-2.07	0.024	4801456	Abcc3	2.91	0.038	4354376	Gstm3	-1.82	0.049	
5342135	Cxcl2	2.72	0.024	4427222	Dmbt1	-4.68	0.038	5410522	Ticam2	-1.76	0.049	
4717379	Krt6a	-3.68	0.024	5429934	Serpinb1c	-3.17	0.038	5124908	Srgn	-3.14	0.049	
5571154	---	2.04	0.024	4594565	Cfi	-2.43	0.038	4677864	ENSMUST00000054308	3.54	0.049	
5221932	Thy1	-3.46	0.024	4862001	Myh9	-2.15	0.039	4918683	Itgax	-1.92	0.050	
*Refers to the difference in the log2 expression of probesets following the three diets in five-week old mice of PTEN null background compared to WT on control diet.
