# Supplementary material for: The dietary isothiocyanate sulforaphane modulates gene expression and alternative gene splicing in a PTEN null preclinical murine model of prostate cancer
Source: Mol Cancer. 2010 Jul 13;9:189. doi: 10.1186/1476-4598-9-189 (PMC3098008; doi:10.1186/1476-4598-9-189)
Supplement: Additional file 8 — Supplementary Table S8. Functional analysis of exons alternatively spliced between eight week old PTEN null mice on low or high SF diets and WT mice on control diet. [file 1476-4598-9-189-S8.RTF]

Supplementary Table S8. Functional analysis of exons alternatively spliced between eight week old PTEN null mice on low or high SF diets and WT mice on control diet.
MAPP Name	Number Changed*	Number Measured**	Adjusted
P-value***	
Low SF diet				
Mm_Ribosomal_Proteins	16	63	<0.001	
Mm_Electron_Transport_Chain-COREG	30	302	<0.001	
Mm_Metabolism-of-glucose-other-sugars-and-ethanol_Reactome-COREG	23	250	0.009	
Mm_Translation_Reactome-COREG	24	272	0.010	
Mm_Translation_Factors-COREG	33	460	0.010	
Mm_Skeletal-muscle-hypertrophy-is-regulated-via-AKT-mTOR-pathway_BioCarta-COREG	20	203	0.010	
Mm_mTOR-Signaling-Pathway_BioCarta-COREG	25	296	0.017	
Mm_Carbon-fixation_KEGG-COREG	19	189	0.017	
Mm_Methionine-metabolism_KEGG-COREG	14	113	0.017	
Mm_Alpha-synuclein-and-Parkin-mediated-proteolysis-in-Parkinsons-disease_BioCarta-MEGINT	2	3	0.017	
High SF diet				
immune response	16	162	0.006	
inflammatory response	10	89	0.027	
Mm_IL-10-Anti-inflammatory-Signaling-Pathway_BioCarta-COREG	3	13	0.115	
Mm_Inflammatory_Response_Pathway	5	35	0.121	
Mm_Inflammatory_Response_Pathway-MEGINT	5	38	0.146	
response to wounding	10	158	0.433	
Mm_Inflammatory_Response_Pathway-COREG	5	50	0.458	
Only pathways with adjusted P-values ≤ 0.05 are shown. Pathway analysis was performed on probesets that were statistically significant (Benjamini and Hochberg adjusted P0.05,) between the two groups. No fold cutoff was used. 
* Refers to the transcripts from the input probeset list identified for each diet that are present in the specific pathway. **Refers to the total number of transcripts that are present in the pathway. ***P-values were calculated in GenMAPP using a non-parametric statistic based on 2000 permutations of the data and further adjusted for multiple testing by Westfall-Young adjustment.
